# DNA methylation signatures of chronic low-grade inflammation are associated with complex diseases

**DOI:** 10.1186/s13059-016-1119-5

**Published:** 2016-12-12

**Authors:** Symen Ligthart, Carola Marzi, Stella Aslibekyan, Michael M. Mendelson, Karen N. Conneely, Toshiko Tanaka, Elena Colicino, Lindsay L. Waite, Roby Joehanes, Weihua Guan, Jennifer A. Brody, Cathy Elks, Riccardo Marioni, Min A. Jhun, Golareh Agha, Jan Bressler, Cavin K. Ward-Caviness, Brian H. Chen, Tianxiao Huan, Kelly Bakulski, Elias L. Salfati, WHI-EMPC Investigators, Giovanni Fiorito, CHARGE epigenetics of Coronary Heart Disease, Simone Wahl, Katharina Schramm, Jin Sha, Dena G. Hernandez, Allan C. Just, Jennifer A. Smith, Nona Sotoodehnia, Luke C. Pilling, James S. Pankow, Phil S. Tsao, Chunyu Liu, Wei Zhao, Simonetta Guarrera, Vasiliki J. Michopoulos, Alicia K. Smith, Marjolein J. Peters, David Melzer, Pantel Vokonas, Myriam Fornage, Holger Prokisch, Joshua C. Bis, Audrey Y. Chu, Christian Herder, Harald Grallert, Chen Yao, Sonia Shah, Allan F. McRae, Honghuang Lin, Steve Horvath, Daniele Fallin, Albert Hofman, Nicholas J. Wareham, Kerri L. Wiggins, Andrew P. Feinberg, John M. Starr, Peter M. Visscher, Joanne M. Murabito, Sharon L. R. Kardia, Devin M. Absher, Elisabeth B. Binder, Andrew B. Singleton, Stefania Bandinelli, Annette Peters, Melanie Waldenberger, Giuseppe Matullo, Joel D. Schwartz, Ellen W. Demerath, André G. Uitterlinden, Joyce B. J. van Meurs, Oscar H. Franco, Yii-Der Ida Chen, Daniel Levy, Stephen T. Turner, Ian J. Deary, Kerry J. Ressler, Josée Dupuis, Luigi Ferrucci, Ken K. Ong, Themistocles L. Assimes, Eric Boerwinkle, Wolfgang Koenig, Donna K. Arnett, Andrea A. Baccarelli, Emelia J. Benjamin, Abbas Dehghan

**Affiliations:** 1 Department of Epidemiology, Erasmus University Medical Center, Rotterdam, The Netherlands; 2 Institute of Epidemiology II, Research Unit of Molecular Epidemiology, Helmholtz Zentrum München, German Research Center for Environmental Health, Neuherber, Germany; 3 German Center for Diabetes Research (DZD e.V.), Partner Munich, Germany; 4 Department of Epidemiology, University of Alabama at Birmingham, Birmingham, AL USA; 5 Boston University School of Medicine, Boston, MA USA; 6 Department of Cardiology, Boston Children’s Hospital, Boston, MA USA; 7 Population Sciences Branch, National Heart, Lung, and Blood Institute, National Institutes of Health, Bethesda, MD USA; 8 Department of Human Genetics, Emory University School of Medicine, Atlanta, GA USA; 9 Translational Gerontology Branch, National Institute on Aging, Baltimore, MD USA; 10 Department of Environmental Health, Harvard T.H. Chan School of Public Health, Boston, MA USA; 11 HudsonAlpha Institute for Biotechnology, Huntsville, AL USA; 12 Hebrew SeniorLife, Harvard Medical School, Boston, MA USA; 13 Division of Biostatistics, School of Public Health, University of Minnesota, Minneapolis, MN USA; 14 Cardiovascular Health Research Unit, Department of Medicine, University of Washington, Seattle, WA USA; 15 MRC Epidemiology Unit, Institute of Metabolic Science, University of Cambridge, Cambridge, UK; 16 Centre for Cognitive Ageing and Cognitive Epidemiology, University of Edinburgh, Edinburgh, UK; 17 Medical Genetics Section, Centre for Genomic and Experimental Medicine, Institute of Genetics and Molecular Medicine, University of Edinburgh, Edinburgh, UK; 18 Queensland Brain Institute, The University of Queensland, Brisbane, QLD Australia; 19 Department of Epidemiology, School of Public Health, University of Michigan, Ann Arbor, MI USA; 20 Human Genetics Center, School of Public Health, University of Texas Health Science Center at Houston, Houston, TX USA; 21 Institute of Epidemiology II, Helmholtz Zentrum München, German Research Center for Environmental Health, Neuherber, Germany; 22 Longitudinal Studies Section, Translational Gerontology Branch, Intramural Research Program, National Institute on Aging, National Institutes of Health, Baltimore, MD USA; 23 Center for Epigenetics, Johns Hopkins University School of Medicine, Baltimore, MD USA; 24 Department of Medicine - Division of Cardiovascular Medicine, Stanford University School of Medicine, Stanford, CA USA; 25 Human Genetics Foundation, Torino, Italy; 26 Department of Medical Sciences, University of Torino, Torino, Italy; 27 Institute of Human Genetics, Helmholtz Center Munich, German Research Center for Environmental Health, Neuherberg, Germany; 28 Institute of Human Genetics, Technical University Munich, München, Germany; 29 Laboratory of Neurogenetics, National Institute on Aging, Bethesda, MD USA; 30 Epidemiology and Public Health, University of Exeter Medical School, RILD Building Level 3 Research, Exeter, UK; 31 Division of Epidemiology & Community Health, School of Public Health, University of Minnesota, Minneapolis, MN USA; 32 Stanford University School of Medicine, Palo Alto, CA USA; 33 VA Palo Alto Health Care System, Palo Alto, CA USA; 34 Department of Biostatistics, Boston University School of Public Health, Boston, MA 02118 USA; 35 Department of Psychiatry and Behavioral Sciences, Emory University School of Medicine, Atlanta, GA USA; 36 Department of Internal Medicine, Erasmus University Medical Center, Rotterdam, The Netherlands; 37 VA Boston Healthcare System and Boston University Schools of Public Health and Medicine, Jamaica Plain, Boston, MA USA; 38 Institute of Clinical Diabetology, German Diabetes Center, Leibniz Center for Diabetes Research at Heinrich Heine University Düsseldorf, Düsseldorf, Germany; 39 German Center for Diabetes Research (DZD e.V.), München-Neuherberg, Germany; 40 University of Queensland Diamantina Institute, Translational Research Institute, The University of Queensland, Brisbane, QLD Australia; 41 UCLA, Department of Human Genetics, Gonda Research Center, David Geffen School of Medicine, Los Angeles, CA USA; 42 Department of Epidemiology, Harvard T.H. Chan School of Public Health, Boston, MA USA; 43 Max-Planck Institute of Psychiatry, Munich, Germany; 44 Geriatric Unit, Azienda Sanitaria Firenze (ASF), Florence, Italy; 45 Department of Environmental Health and Epidemiology, Harvard T.H. Chan School of Public Health, Boston, MA USA; 46 Department of Pediatrics, Harbor-UCLA Medical Center, Torrance, CA USA; 47 Division of Nephrology and Hypertension, Mayo Clinic, Rochester, MN USA; 48 Division of Depression & Anxiety Disorders, McLean Hospital, Belmont, MA USA; 49 Department of Psychiatry, Harvard Medical School, Boston, MA USA; 50 Department of Medicine, Stanford University School of Medicine, Stanford, CA USA; 51 Department of Internal Medicine II-Cardiology, University of Ulm Medical Center, Ulm, Germany; 52 Deutsches Herzzentrum München, Technische Universität München, Munich, Germany; 53 DZHK (German Center for Cardiovascular Research), partner site Munich Heart Alliance, Munich, Germany; 54 University of Kentucky, College of Public Health, Lexington, KY USA; 55 Department of Epidemiology, Boston University School of Public Health, Boston, MA USA; 56 Boston University and the NHLBI’s Framingham Heart Study, Boston, MA USA; 57 Department of Biostatistics and Epidemiology, MRC-PHE Centre for Environment and Health, School of Public Health, Imperial College London, London, UK

**Keywords:** Inflammation, DNA methylation, Epigenome-wide association study, C-reactive protein, Body mass index, Diabetes, Coronary heart disease

## Abstract

**Background:**

Chronic low-grade inflammation reflects a subclinical immune response implicated in the pathogenesis of complex diseases. Identifying genetic loci where DNA methylation is associated with chronic low-grade inflammation may reveal novel pathways or therapeutic targets for inflammation.

**Results:**

We performed a meta-analysis of epigenome-wide association studies (EWAS) of serum C-reactive protein (CRP), which is a sensitive marker of low-grade inflammation, in a large European population (*n* = 8863) and trans-ethnic replication in African Americans (*n* = 4111). We found differential methylation at 218 CpG sites to be associated with CRP (*P* < 1.15 × 10^–7^) in the discovery panel of European ancestry and replicated (*P* < 2.29 × 10^–4^) 58 CpG sites (45 unique loci) among African Americans. To further characterize the molecular and clinical relevance of the findings, we examined the association with gene expression, genetic sequence variants, and clinical outcomes. DNA methylation at nine (16%) CpG sites was associated with whole blood gene expression in cis (*P* < 8.47 × 10^–5^), ten (17%) CpG sites were associated with a nearby genetic variant (*P* < 2.50 × 10^–3^), and 51 (88%) were also associated with at least one related cardiometabolic entity (*P* < 9.58 × 10^–5^). An additive weighted score of replicated CpG sites accounted for up to 6% inter-individual variation (R2) of age-adjusted and sex-adjusted CRP, independent of known CRP-related genetic variants.

**Conclusion:**

We have completed an EWAS of chronic low-grade inflammation and identified many novel genetic loci underlying inflammation that may serve as targets for the development of novel therapeutic interventions for inflammation.

**Electronic supplementary material:**

The online version of this article (doi:10.1186/s13059-016-1119-5) contains supplementary material, which is available to authorized users.

## Background

Chronic low-grade inflammation is a complex immune response that plays an important role in the pathogenesis of multiple chronic diseases, including diabetes and cardiovascular disease [1, 2]. C-reactive protein (CRP) is a sensitive marker of chronic low-grade inflammation in community-dwelling adults [3] and is associated in population-based studies with an increased risk of incident coronary heart disease (CHD), stroke, and non-vascular mortality [4]. Several pathways have been identified for chronic low-grade inflammation [1, 5] and genetic studies have found candidate loci through discovery of genetic sequence determinants of circulating CRP levels [6]. However, most of the molecular mechanisms underlying inter-individual variation in inflammation in the general population and the inter-relation with complex diseases remain to be elucidated.

Epigenetic modifications comprise biochemical alterations to the genome that leave the underlying nucleic acid sequence unchanged but can affect phenotypic expression. DNA methylation is a pivotal and stable epigenetic mechanism whereby a methyl group is attached to the DNA sequence, most often a cytosine nucleotide that neighbors a guanine nucleotide. DNA methylation is affected by both genetic and environmental factors and regulates gene expression and chromosome stability [7]. Investigating DNA methylation in chronic low-grade inflammation may point to functional epigenetic changes that occur in the context of inflammation.

**Fig. 1:**
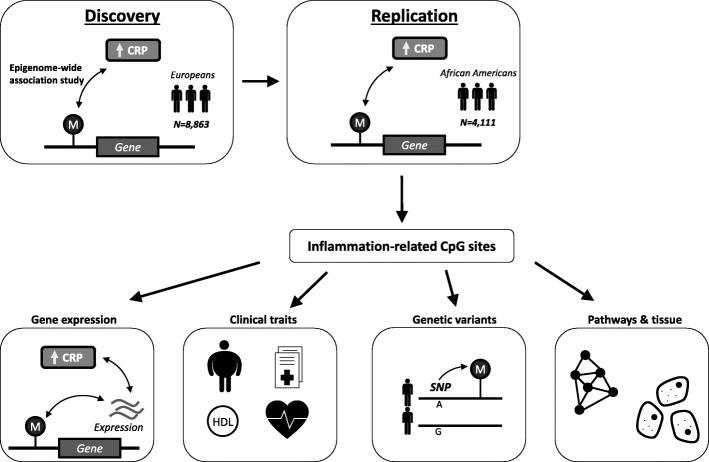
*Illustration* of overall study flow

We performed the first meta-analysis of epigenome-wide association studies (EWAS) of methylation of DNA on chronic low-grade inflammation using CRP as a sensitive inflammatory biomarker (Fig. 1). We first conducted a discovery meta-analysis, comprising 8863 participants of European ancestry. Since race or ethnicity may affect epigenetic associations [8], we conducted trans-ethnic replication in 4111 individuals of African-American ancestry. We further investigated the association between replicated DNA methylation sites and both *cis*- gene expression and genetic variants. Finally, differentially methylated CpG sites were examined for association with cardiometabolic phenotypes to study potential epigenetic links between inflammation and cardiometabolic diseases.

## Results

### Clinical characteristics

The nine participating discovery (*n* = 8863) and four replication cohorts (*n* = 4111) and the clinical characteristics of the participants are presented in Table 1 (further details, Additional file 1: Table S1). The mean age in the participating studies ranged from 41 years in the Grady Trauma Project (GTP) cohort to 87 years in Lothian Birth Cohort (LBC) 1921. The majority (54%) of the samples were from women. Some of the cohorts differed based on selection criteria for entry into the study. The Normative Aging Study (NAS) only included men, while the Women’s Health Initiative (WHI) only included women. Mean serum CRP levels (SD) ranged from 2.3 (3.7) mg/L in the Kooperative Gesundheitsforschung in der Region Augsburg (KORA) study to 7.2 (8.4) mg/L in the African-American CHD cases of WHI.

**Table 1:** Characteristics of the discovery (*n* = 8863) and replication (*n* = 4111) studies

Study	n	Country	Age (years)	Women (%)	CRP (mg/L)	BMI (kg/m^2^)
Discovery (European)						
CHS	187	USA	76 (5)	56	6.6 (11.0)	31 (6)
EPIC-Norfolk	1287	UK	60 (9)	54	3.3 (5.4)	27 (4)
FHS	2427	USA	66 (9)	52	3.1 (6.7)	28 (5)
InCHIANTI	498	Italy	63 (16)	55	3.2 (3.5)	27 (4)
KORA	1700	Germany	61 (9)	51	2.3 (3.7)	28 (5)
LBC 1921	169	UK	87 (0)	54	3.7 (8.4)	26 (4)
LBC 1936	296	UK	70 (1)	50	5.3 (6.8)	28 (4)
NAS	648	USA	73 (7)	0	3.3 (6.1)	28 (4)
Rotterdam	702	Netherlands	60 (8)	54	2.7 (4.7)	28 (5)
WHI controls	471	USA	68 (6)	100	3.8 (5.5)	28 (6)
WHI cases	478	USA	69 (6)	100	4.9 (6.4)	29 (6)
Replication (African American)						
ARIC	2264	USA	56 (6)	64	5.9 (7.8)	30 (6)
CHS	193	USA	73 (5)	65	5.2 (5.6)	29 (5)
GENOA	939	USA	66 (8)	71	6.7 (12.3)	31 (6)
GTP	112	USA	41 (13)	70	5.9 (8.1)	33 (8)
WHI controls	309	USA	62 (6)	100	6.1 (7.5)	31 (7)
WHI cases	294	USA	64 (7)	100	7.2 (8.4)	32 (6)

Characteristics are mean (SD), unless otherwise specified

*ARIC* Atherosclerosis Risk in Communities, *BMI* body mass index, *CHS* Cardiovascular Health Study, *CRP* C-reactive protein, *EPIC-Norfolk* European Prospective Investigation into Cancer and Nutrition Norfolk, *FHS* Framingham Heart Study, *GENOA* Genetic Epidemiology Network of Arteriopathy, *InCHIANTI* Invecchiare in Chianti, *KORA* Kooperative Gesundheitsforschung in der Region Augsburg, *LBC* Lothian Birth Cohort, *NAS* Normative Aging Study, *UK* United Kingdom, *USA* United States of America, *WHI* Women’s Health Initiative

### Discovery meta-analysis

**Fig. 2:**
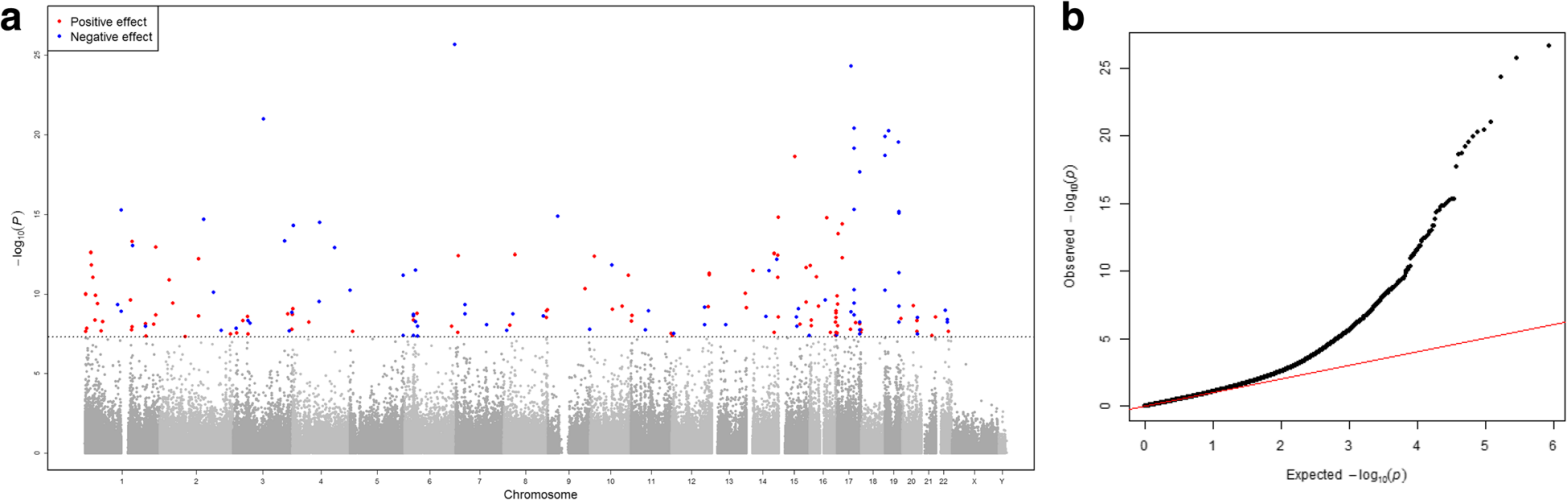
**a** *Manhattan plot* depicting the –log_10_(*P* values) of the associations between all CpG sites and CRP, adjusted for age, sex, BMI, cell distributions, technical covariates, and smoking. The *dotted lines* indicate the Bonferroni threshold of 1.15 × 10^−7^ for significance. The *red dots* indicate positive significant associations between methylation and CRP, the *blue dots* indicate negative significant associations. **b** *QQ plot* showing observed vs. expected − log_10_(*P* values) for association at all CpG sites

We identified 218 CpG sites significantly associated (*P* < 1.15 × 10^−7^) with CRP in the meta-analysis of European participants, adjusted for age, sex, white blood cell proportions, technical covariates, smoking, and body mass index (BMI) (Manhattan and QQ-plot, Fig. 2, Additional file 2: Table S2, and Additional file 3: Table S3). Serum CRP was positively associated with 125 CpG sites and negatively associated with 93. The top CpG site was cg10636246 at 1q23.1 located within 1500 bp of the transcription start site of *Absent in melanoma 2* (*AIM2*) (effect size = −0.0069, *P* = 2.53 × 10^−27^), an interferon-gamma-induced protein involved in the innate immune response by inducing caspase-1-activating inflammasome formation in macrophages.

### Replication meta-analysis

Of the 218 CpG sites significantly associated with CRP in our discovery meta-analysis, 58 replicated (*P* < 2.29 × 10^−4^) in a trans-ethnic replication meta-analysis of 4111 individuals of African-American ancestry (Table 2). The replicated CpG sites annotated to 45 separate loci. The most significant CpG site in the discovery panel (cg10636246; *AIM2*) was also strongly related to serum CRP in individuals of African-American ancestry (effect size = −0.0081, *P* = 6.31 × 10^−9^). Effect estimates of the 58 replicated CpG sites assessed in the European and African-American panel were highly correlated (*r* = 0.97). Cochrane’s Q statistics displayed homogeneity for > 95% of the 58 replicated loci in both the European discovery panel and the African-American replication panel (study specific effect estimates, Additional file 4). In addition, we conducted a meta-analysis combining the European and African-American whole blood samples resulting in 258 significant CpGs (Additional file 5).

**Table 2:** DNA methylation sites associated with serum CRP levels

CpG sites	Chr	Position	Effect size EA	*P* value EA	Effect size AA	P value AA	Gene
cg10636246	1	159046973	−0.0069	2.53 × 10^−27^	−0.0081	6.31 × 10^−09^	*AIM2*
cg17501210	6	166970252	−0.0065	2.06 × 10^−26^	−0.0076	9.45 × 10^−05^	*RPS6KA2*
cg02650017	17	47301614	−0.0021	4.87 × 10^−25^	−0.0011	7.71 × 10^−06^	*PHOSPHO1*
cg12992827	3	101901234	−0.0057	9.73 × 10^−22^	−0.0086	4.42 × 10^−14^	*NFKBIZ*
cg16936953	17	57915665	−0.0077	3.74 × 10^−21^	−0.0125	1.13 × 10^−13^	*TMEM49*
cg19821297	19	12890029	−0.0051	5.19 × 10^−21^	−0.0055	6.58 × 10^−06^	*GCDH*
cg07573872	19	1126342	−0.0052	1.24 × 10^−20^	−0.0068	2.98 × 10^−09^	*SBNO2*
cg26470501	19	45252955	−0.0045	2.85 × 10^−20^	−0.0051	4.08 × 10^−07^	*BCL3*
cg12054453	17	57915717	−0.0082	6.96 × 10^−20^	−0.0117	4.25 × 10^−12^	*TMEM49*
cg18608055	19	1130866	−0.0043	1.94 × 10^−19^	−0.0078	2.96 × 10^−11^	*SBNO2*
cg06192883	15	52554171	0.0045	2.29 × 10^−19^	0.0073	8.29 × 10^−12^	*MYO5C*
cg18181703	17	76354621	−0.0053	2.13 × 10^−18^	−0.0091	7.08 × 10^−13^	*SOCS3*
cg18942579	17	57915773	−0.0056	4.77 × 10^−16^	−0.0098	8.70 × 10^−12^	*TMEM49*
cg19769147	14	105860954	0.0029	1.51 × 10^−15^	0.0029	6.60 × 10^−05^	*PACS2*
cg20995564	2	145172035	−0.0051	2.04 × 10^−15^	−0.0089	2.69 × 10^−10^	*ZEB2*
cg02734358	4	90227074	−0.0048	3.09 × 10^−15^	−0.0051	5.51 × 10^−05^	*GPRIN3*
cg07094298	4	2748026	−0.0056	4.76 × 10^−15^	−0.0058	5.32 × 10^−06^	*TNIP2*
cg01059398	3	172235808	−0.0042	4.51 × 10^−14^	−0.0068	2.27 × 10^−05^	*TNFSF10*
cg06690548	4	139162808	−0.0048	1.21 × 10^−13^	−0.0029	1.52 × 10^−07^	*SLC7A11*
cg02003183	14	103415882	0.0047	3.59 × 10^−13^	0.0051	4.36 × 10^−05^	*CDC42BPB*
cg26804423	7	8201134	0.0027	3.87 × 10^−13^	0.0038	4.82 × 10^−07^	*ICA1*
cg13585930	10	72027357	−0.0037	1.42 × 10^−12^	−0.0046	7.95 × 10^−05^	*NPFFR1*
cg03957124	6	37016869	−0.0030	3.13 × 10^−12^	−0.0039	1.39 × 10^−05^	*FGD2*
cg12053291	12	125282342	0.0029	5.99 × 10^−12^	0.0038	9.80 × 10^−05^	*SCARB1*
cg02481950	16	21665002	0.0022	7.84 × 10^−12^	0.0034	2.92 × 10^−06^	*METTL9*
cg04987734	14	103415873	0.0041	8.40 × 10^−12^	0.0051	1.40 × 10^−04^	*CDC42BPB*
cg15551881	9	123688715	0.0039	4.62 × 10^−11^	0.0049	3.99 × 10^−07^	*TRAF1*
cg27023597	17	57918262	−0.0050	5.02 × 10^−11^	−0.0070	5.96 × 10^−06^	*MIR21*
cg05575921	5	373378	−0.0059	5.44 × 10^−11^	−0.0063	1.17 × 10^−04^	*AHRR*
cg27469606	19	1154485	−0.0020	5.62 × 10^−11^	−0.0023	1.96 × 10^−06^	*SBNO2*
cg01409343	17	57915740	−0.0037	3.56 × 10^−10^	−0.0081	6.12 × 10^−10^	*TMEM49*
cg21429551	7	30635762	−0.0069	4.42 × 10^−10^	−0.0080	1.68 × 10^−05^	*GARS*
cg23761815	10	73083123	0.0022	8.86 × 10^−10^	0.0029	6.85 × 10^−05^	*SLC29A3*
cg08548559	22	31686097	−0.0038	9.94 × 10^−10^	−0.0049	9.88 × 10^−05^	*PIK3IP1*
cg26610247	8	142297175	0.0029	1.07 × 10^−09^	0.0041	4.59 × 10^−06^	*TSNARE1*
cg27050612	17	46133198	−0.0019	1.30 × 10^−09^	−0.0029	8.23 × 10^−05^	*NFE2L1*
cg15721584	3	181326755	0.0055	1.71 × 10^−09^	0.0072	1.14 × 10^−05^	*SOX2OT*
cg06126421	6	30720080	−0.0052	1.80 × 10^−09^	−0.0059	1.53 × 10^−04^	*TUBB*
cg00851028	1	234905772	0.0023	1.95 × 10^−09^	0.0042	1.46 × 10^−05^	*-*
cg24174557	17	57903544	−0.0038	1.97 × 10^−09^	−0.0051	1.65 × 10^−04^	*TMEM49*
cg05316065	8	130799007	−0.0027	2.26 × 10^−09^	−0.0051	2.28 × 10^−07^	*GSDMC*
cg04523589	3	48265146	0.0022	2.49 × 10^−09^	0.0031	4.47 × 10^−05^	*CAMP*
cg17980786	3	32933637	0.0026	4.58 × 10^−09^	0.0055	1.47 × 10^−09^	*TRIM71*
cg25325512	6	37142220	−0.0031	5.31 × 10^−09^	−0.0052	4.94 × 10^−05^	*PIM1*
cg00812761	4	53799391	0.0025	5.60 × 10^−09^	0.0036	1.36 × 10^−04^	*SCFD2*
cg27637521	17	76355202	−0.0016	5.69 × 10^−09^	−0.0017	3.69 × 10^−05^	*SOCS3*
cg26846781	17	61620942	0.0018	5.99 × 10^−09^	0.0033	3.03 × 10^−05^	*KCNH6*
cg00159243	12	109023799	−0.0026	8.22 × 10^−09^	−0.0036	1.38 × 10^−04^	*SELPLG*
cg15310871	8	20077936	0.0022	8.63 × 10^−09^	0.0027	2.96 × 10^−05^	*ATP6V1B2*
cg15020801	17	46022809	0.0024	1.67 × 10^−08^	0.0033	9.47 × 10^−05^	*PNPO*
cg03128029	2	203143288	−0.0027	1.90 × 10^−08^	−0.0036	2.03 × 10^−04^	*NOP58*
cg22749855	17	76353952	−0.0024	3.22 × 10^−08^	−0.0035	5.15 × 10^−05^	*SOCS3*
cg02341197	21	34185927	0.0030	3.92 × 10^−08^	0.0045	2.54 × 10^−05^	*C21orf62*
cg12269535	6	43142014	−0.0028	4.39 × 10^−08^	−0.0046	1.57 × 10^−04^	*SRF*
cg25392060	8	142297121	0.0025	5.60 × 10^−08^	0.0036	2.15 × 10^−04^	*TSNARE1*
cg27184903	15	29285727	0.0024	5.84 × 10^−08^	0.0052	4.91 × 10^−07^	*APBA2*
cg18663307	21	46341389	0.0029	6.98 × 10^−08^	0.0048	1.04 × 10^−04^	*ITGB2*
cg09182678	22	50328711	−0.0016	9.02 × 10^−08^	−0.0019	1.26 × 10^−04^	*DENND6B*

Effect sizes represent the changes in normalized DNA methylation Beta-values per 1-unit increase in natural log-transformed CRP (mg/L)

Chr and Position are in GRCh37/hg19

*AA* African American, *EA* European Ancestry

### Sensitivity analyses

Further adjustment of the replicated CpG sites for additional potential confounders (waist circumference, total/HDL-cholesterol ratio, prevalent diabetes, hypertension treatment, lipid treatment, hormone replacement therapy, and prevalent CHD) did not substantially change the effect estimates and *P* values. Additional file 6: Figure S3 depicts the correlation between the effect estimates and –log10 *P* values in the primary model compared to the multivariable adjusted model, respectively. Furthermore, 18 CpGs were found to be associated with serum CRP levels in CD4+ cells in the GOLDN study (*P* < 0.05) (Additional file 7: Table S6).

### Methylation and genetic scores

Additive weighted methylation and genetic scores were constructed to calculate percentage of total CRP variance explained. A methylation score including eight independent CpGs (cg10636246, cg17501210, cg18608055, cg03957124, cg04987734, cg04523589, cg17980786, and cg02341197) explained 5.8% of the variance of CRP in Atherosclerosis Risk in Communities (ARIC), 2.3% in KORA, 5.0% in NAS, and 4.6% in RS. A genetic score including 18 independent CRP single nucleotide polymorphisms (SNPs) explained 4.9% of the CRP variance in RS and the methylation and genetic scores together explained 9.0%. Notably, no significant interaction or association was observed between the genetic and methylation scores, suggesting that they independently explain variance in CRP.

### Association with cardiometabolic phenotypes

**Fig. 3:**
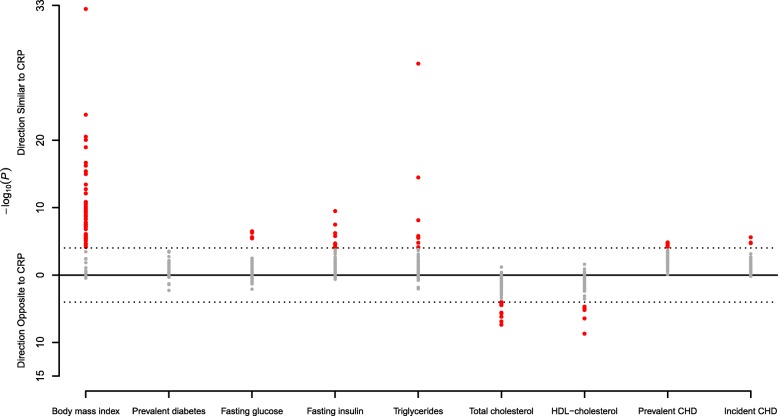
*Manhattan plot* depicting the –log_10_(*P* values) and effect direction (respectively to CRP) of the associations between the 58 replicated CpG sites and each cardiometabolic phenotype, adjusted for age, sex, BMI, cell distributions, technical covariates, and smoking. The *dotted lines* indicate the Bonferroni threshold of 9.58 × 10^−5^ for significance

We examined the associations between the 58 replicated CRP-related CpG sites and nine cardiometabolic traits and diseases (BMI, lipids, glycemic phenotypes, prevalent CHD, and incident CHD). After Bonferroni correction for multiple testing based on 58 CpG sites and nine phenotypes (*P* < 0.05/522 = 9.58 × 10^−5^), we observed 89 significant associations with 51 unique CpG sites (Additional file 8: Table S7). There was major overlap with BMI (46 CpGs). CpGs that were significantly associated with higher BMI, fasting glucose, fasting insulin, risk of diabetes, triglycerides, and risk of CHD were also associated with higher CRP levels. For HDL-cholesterol and total cholesterol, CpGs were associated with lower CRP levels (Fig. 3).

### Gene expression analyses

**Fig. 4:**
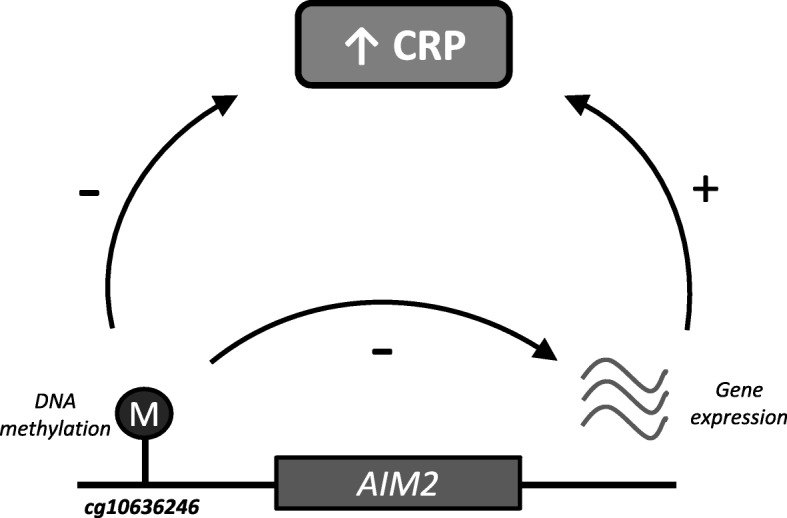
*Illustration* of the methylation-CRP, methylation-expression, and expression-CRP association for cg10636246 (*AIM2*)

Of the 58 replicated CpG sites, nine (16%) were significantly associated with expression of nine unique genes in *cis* (*P* < 8.47 × 10^−5^) (Additional file 9: Table S8). Furthermore, of those nine genes, the expression levels of four genes were associated with serum CRP levels (*P* < 0.05). In these four cases, we could show corresponding triangular relationships between DNA methylation, gene expression, and serum CRP levels. For example, increased methylation at cg10636246 was associated with lower serum CRP levels and lower expression of *AIM2* and lower expression of *AIM2* was associated with lower CRP levels (Fig. 4).

### Genetic correlates of DNA methylation in cis

In the RS, we identified 20 *cis*-mQTL pairs (19 unique SNPs and 20 unique CpG sites) for the replicated CpG sites, ten of these cis-mQTL pairs could be replicated in the Framingham Heart Study (FHS) (*P* < 2.5 × 10^−3^) (Additional file 10: Table S9). For example, the strongest correlation was observed between rs12677618 and cg25392060 (located 4903 bp away from each other; β = −0.011; *P* = 2.73 × 10^−126^). None of the ten replicated *cis-*mQTL variants was significantly associated with serum CRP levels after Bonferroni correction for multiple testing (*P* > 0.005) in the largest published genome-wide association study (GWAS) to date of 66,185 individuals [6].

### GWAS catalog, pathway analysis, and tissue enrichment

The 58 CpG sites were annotated to 47 genes, which are associated in GWAS with 18 phenotypes (Additional file 11: Table S10). We found enrichment in GWAS of epilepsy, renal cell carcinoma, and lipoprotein-associated phospholipase A2 (Lp-PLA2) activity and mass.

Pathway enrichment analyses were carried out in 47 unique genes that were annotated to the 58 replicated CpG sites in the Ingenuity Pathway Analysis (IPA) database. The top pathways included growth hormone signaling, IL-9 signaling, atherosclerosis, and IL-6 signaling (Additional file 12: Table S11).

Analysis of tissue specific DNase I hotspots yielded enrichment predominantly in epithelium, blood vessels, and various blood cells (especially CD14+ macrophages) (Additional file 6: Table S4).

## Discussion

This meta-analysis of EWAS of CRP, a sensitive marker of chronic low-grade inflammation, identified and validated 58 CpG sites in or near 45 unique loci in leukocytes of individuals of European and African descent. The associations were robust to adjustment for potential confounders and explained more than 6% of the variation in circulating CRP concentrations. We demonstrated that several inflammation-related CpG sites were associated with expression of nearby genes and many CpG sites showed pleiotropic associations with cardiometabolic phenotypes as well as the clinical disease CHD.

DNA methylation may differ by race or ethnicity [8], challenging replication across individuals of varying descent in epigenetic studies. We were able to replicate up to 27% of our findings with comparable effect estimates, demonstrating that our results are generalizable across Europeans and African Americans. The trans-ethnic replication approach of our study strengthens the confidence of true-positive findings and supports the notion that despite differing baseline epigenetic profiles, different ethnicities may have consistent epigenetic associations with respect to inflammation.

Increased DNA methylation at the top signal cg10636246 near *AIM2* was associated with lower expression of *AIM2* and lower CRP levels. In agreement, lower *AIM2* expression was associated with lower serum CRP levels. As an inflammasome receptor for double-stranded DNA activating inflammatory cascades, *AIM2* is implicated in host defense mechanisms against bacterial and viral pathogens and thus is key in the human innate immune response [9, 10]. The data suggest that methylation near *AIM2* plays a role in low-grade inflammation in the general population. Nevertheless, the results from the current study do not infer causal directionality.

Several of our hits were associated with future clinical events. For example, three inflammation-related CpG sites were also associated with incident CHD. Hypomethylation at cg18181703 (*SOCS3)*, cg06126421 (*TUBB*), and cg05575921 (*AHRR*) were associated with higher CRP levels and increased risk of future CHD. The gene product of *SOCS3*, suppressor of cytokine signaling 3, plays a pivotal role in the innate immune system as a regulator of cytokine signaling [11]. The role of *SOCS3* in atherosclerosis has been established [12]. We observed that lower DNA methylation was associated with increased expression of *SOCS3* and increased serum CRP. Differential methylation at the *AHRR* loci has been robustly demonstrated to be associated with cigarette smoking [13]. The association of *AHRR* methylation with CRP and incident CHD may highlight a connection between CRP and cardiovascular disease that is shared between cigarette smoking and independent mechanisms. Furthermore, we found two CpG sites that have recently been identified in an EWAS of incident type 2 diabetes [14]. We hypothesize that inflammation-related epigenetic features may explain at least part of the observed associations between CRP, a sensitive marker of chronic low-grade inflammation, and related clinical events including CHD and diabetes.

Many replicated CpG sites demonstrated associations with cardiometabolic phenotypes, emphasizing the substantial epigenetic overlap with those phenotypes. Taken together, these pleiotropic epigenetic associations across various phenotypes may provide novel insights into shared epigenetic mechanisms and provide opportunities to link chronic low-grade inflammation and cardiometabolic phenotypes. Our findings may help to focus on genomic regulation of pertinent loci that may be attractive targets for perturbation or therapeutic intervention.

CRP is affected by both genetic and environmental factors [15]. Although we may have slightly overestimated the variance explained since the testing cohorts participated in the discovery and replication meta-analysis, the CRP methylation score augmented the explained variance beyond that accounted for by the CRP genetic score. This suggests that the methylation score harbors information that may be independent from the genetic factors underlying CRP. In agreement with a previous report on the added value of a methylation score in explaining variance in BMI, we further add that methylation may explain further variation of complex traits that have substantial environmental components [16].

In the present study, we were able to present stringent triangular relationships between DNA methylation, gene expression, and serum CRP levels at four loci. However, firm conclusions regarding causal directionality are challenging in epigenetic studies. Although ten (17%) of the replicated methylation sites had *cis-*mQTLs, we were not able to detect a significant association between these mQTLs and CRP levels in the largest published CRP GWAS, which may be due to the limited power, or the findings represent methylation changes downstream of CRP. However, our findings were biologically plausible and consistent with previous observations. For example, GWAS enrichment analysis suggested enrichment in genes identified for renal cell carcinoma. CRP is commonly elevated in renal cell carcinoma patients [17]. Furthermore, pathway analyses identified regulatory mechanisms related to inflammatory processes such as STAT3 and IL-6 signaling pathway, the pro-inflammatory upstream regulator of serum CRP levels [18]. Taken together, these results suggest that DNA methylation plays a role in establishing or maintaining CRP levels in the general population.

The major strengths of the present study are its large sample size and multi-ethnic nature, allowing a valid interpretation of results for both European and African-American populations. Furthermore, careful and comprehensive adjusting models reduced the chance of confounding. In addition, DNA methylation was quantified in whole blood, which is primarily composed of leukocytes, a key component of the human immune system and therefore highly relevant to systemic inflammation. The combination of epigenomics with genomics and transcriptomics data as well as enrichment analyses allowed the exploration of functional properties of our findings.

The study has limitations. The 450 K array captures approximately 2–4% of the total human DNA methylation, mainly in genic regions, thus limits the discovery of potentially important CpG sites that are not measured on the array. Furthermore, although we adjusted the analyses for measured or estimated cell type proportions, we cannot completely rule out the presence of residual confounding by white blood cell distributions. Residual confounding from differences in unmeasured cell count heterogeneity introduced by correlation between CRP and unknown cell subtypes may bias our results. Also, the annotation of CpGs and SNPs to genes is challenging in genomic studies. We annotated primarily based on distances, which may have incorrectly annotated genes. Further, we replicated our findings from the European discovery in African Americans. The differences in ethnicities and the African-American sample size may have limited replication of the findings. Our study was limited to blood samples and while this has been demonstrated to be a good surrogate tissue [19], we would not be able to infer tissue specific methylation changes. Specifically, as CRP is synthesized in the liver, our current study design would not allow us to detect hepatic methylation changes. We did not observe associations with nearby gene expression for all CpGs we identified. However, the limited sample size for methylation-expression analyses, failure for expression probes to pass quality control, tissue-specificity, and long-distance effects may explain this observation. Furthermore, DNA methylation may also affect chromosome stability and alternative splicing, two functional consequences of DNA methylation which we have not investigated in the present study. Finally, we cannot exclude residual confounding and cannot determine causal directionality.

## Conclusions

We performed the first meta-analysis of EWAS of CRP, a sensitive marker of low-grade inflammation. We identified 58 DNA methylation sites that are significantly associated with CRP levels in individuals of both European and African-American ancestry. Since inflammation is implicated in the development of multiple complex diseases, the discoveries from the current study may contribute to the identification of novel therapies and interventions for treatment of inflammation and its clinical consequences.

## Methods

### Discovery and replication study population

Our study was conducted within the framework of the Epigenetics working group of the Cohorts for Heart and Aging Research in Genomic Epidemiology (CHARGE) consortium [20]. The discovery study population comprised 8863 individuals from the following 11 cohort studies (listed in alphabetical order): the Cardiovascular Health Study (CHS), the European Prospective Investigation into Cancer and Nutrition (EPIC) Norfolk study, the FHS, the Invecchiare in Chianti study (InCHIANTI), the KORA study, the LBCs 1921 and 1936 (LBC1921/1936), the NAS, the Rotterdam Study (RS), and the WHI. All individuals in the discovery cohorts were of European descent. The trans-ethnic replication population consisted of 4111 African-American individuals from the ARIC study, the CHS, the Genetic Epidemiology Network of Arteriopathy (GENOA) study, the GTP, and the WHI. The studies are described in detail in Additional file 13: Supplemental methods. Individuals with autoimmune diseases (rheumatoid arthritis, lupus erythematosus, Crohn’s disease, type 1 diabetes) and individuals receiving immune-modulating agents were excluded from all analyses, when disease status and medication data were available. Individuals without such data were assumed to be disease-free and non-users. All participants gave written informed consent and protocols were approved by local institutional review boards and ethic committees.

### C-reactive protein measurements

Serum CRP was measured in mg/L using high-sensitivity assays in all studies except the LBCs, in which CRP was measured with the use of a normal sensitivity assay. CRP was measured in blood samples drawn at the same time and center visit as blood was drawn for DNA methylation quantification. CRP values were natural log-transformed (lnCRP). Study-specific methods on the quantification of CRP are described in Additional file 13: Supplemental methods. Distributions of the natural log-transformed serum CRP levels per study are depicted in Additional file 6: Figure S1.

### DNA methylation quantification

For the quantification of the DNA methylation, DNA was extracted from whole blood in all studies. All studies used the Illumina Infinium Human Methylation450K BeadChip (Illumina Inc, San Diego, CA, USA) for DNA methylation measurement except GENOA, which used the Illumina Infinium HumanMethylation27K BeadChip (Illumina Inc, San Diego, CA, USA). The 450 K Beadchip assays methylation of > 480,000 CpGs and is enriched for gene regions and covers 99% of all genes. DNA methylation data pre-processing was conducted independently in different studies and β values were normalized using study-specific methods. We used methylation β values to represent the proportion of the total signal intensity, which is in the range of 0–1. Further study-specific methods and filtering criteria can be found in Additional file 13: Supplemental methods and Additional file 2: Table S2. A CpG site was deemed polymorphic when a SNP in the 1000 Genomes Project (Phase 1) with a minor allele frequency ≥0.01 resided at the position of the cytosine or guanine on either strand, or within 10 bp from the CpG within the probe binding site [8]. Polymorphic CpG sites were excluded from all analyses. Also, cross-reactive probes were excluded from all analyses [21]. In total, 434,253 probes were available for analysis.

### Epigenome-wide association study

The EWAS was performed at each center separately. Individuals with CRP values > 4 standard deviations (SD) from the respective cohort mean lnCRP were excluded from all analyses. In the primary model, we used linear mixed effect regression models to study the methylation β-values, specified as the dependent variable, as a function of lnCRP adjusting for age, sex, white blood cell proportions, technical covariates (array number and position on array), smoking (current, former and never), and BMI. Technical covariates were modeled as random effects. Measured or estimated (Houseman method implemented in the *minfi* package in R [22, 23]) leukocyte proportions were included to account for cell type admixture (Additional file 2: Table S2). When applicable, models were additionally adjusted for study specific covariates such as study site (fixed effect) and family structure (random effect). Regression models and adjustments were comparable in the discovery and replication analyses. The effect size represents the change in DNA methylation per 1-unit increase in lnCRP.

### Meta-analysis

Fixed effects meta-analyses were conducted using the inverse-variance weighted method implemented in METAL, corrected for double lambda control (individual studies and meta-analysis) [24]. In the discovery phase, a Bonferroni correction was applied to correct for multiple testing with a significance threshold of 0.05/434,253 = 1.15 × 10^−7^. We then examined the significant CpG sites for trans-ethnic replication in 4111 individuals of African-American ancestry using a Bonferroni-corrected significance threshold for the number of CpG sites taken forward for replication. Between-study heterogeneity was examined with Cochran’s Q statistic with a Bonferroni-corrected significance threshold for the number of replicated CpG sites. We performed a power calculation for the replication analysis using the GPower 3.1 tool (Additional file 6: Figure S2) [25]. Additionally, the European and African-American samples were combined in one meta-analysis.

### Sensitivity analyses

In a subset of the discovery cohorts that had further confounders available (CHS, FHS, InCHIANTI, KORA, NAS, RS, and WHI), the replicated CpG sites were additionally adjusted for other potential confounders. These covariates were selected based on strong associations with CRP in observational research [15]. In addition to the variables of the primary model, the sensitivity model included waist circumference, total/high-density lipoprotein (HDL)-cholesterol ratio, prevalent diabetes (defined as fasting glucose ≥7.0 mmol/L, non-fasting glucose ≥11.1 mmol/L, or the use of diabetes medication), hypertension treatment (use of diuretics, anti-adrenergic agents, β-blockers, calcium channel blockers, and RAAS inhibitors), lipid treatment (use of statins, ezetimibe, and colestyramine), hormone replacement therapy, and prevalent CHD. Since the population for analysis in the second model was expected to be slightly smaller compared to the primary model due to missing data for certain covariates, we repeated the primary model to include only individuals present in the second model.

To investigate the association between the replicated CpG sites and serum CRP levels in CD4+ cells, we tested the association in the Genetics of Lipid Lowering Drugs and Diet Network (GOLDN) study which quantified DNA methylation in CD4+ cells. Associations with a consistent effect direction and *P* < 0.05 were considered significant.

### Annotation of CpG sites

We used the genome coordinates provided by Illumina (GRCh37/hg19) to identify independent loci. A distance criterion of 500 kb on either side of each epigenome-wide significant signal was used to define independent loci. In addition to the gene annotation provided by Illumina based on RefSeq database, the UCSC database was explored to further annotate the CpG sites to potential genes (nearest gene).

### Methylation and genetic score

To calculate the variance explained by the replicated CpGs, we first selected independent CpGs based on pairwise Pearson correlation R^2^. To this end, we first ranked the significant CpGs by discovery *P* value in ascending order. We then iteratively excluded CpGs correlated with the top CpG site (*r* ^*2*^ > 0.1) until we reached a list of independent CpGs (*n* = 8). The eight CpGs were used to construct a methylation score weighted by the effect estimates from regression in the FHS with lnCRP as the dependent variable and residuals of the DNA methylation (after regressing out age, sex, batch effect, cell counts, smoking, and BMI) as the independent variable. Using a linear regression model, we calculated the CRP variance explained by the methylation score (multiple R^2^, adjusting for age and sex) in ARIC, KORA, NAS, and RS. Furthermore, an additive effect-size weighted genetic score for CRP was constructed in RS to include 18 SNPs identified in the largest GWAS of CRP (genotyping information RS in Additional file 13: Supplemental methods) [6]. We calculated weighted dosages by multiplying the dosage of each risk allele (0, 1, or 2) with the published effect estimate. We calculated the CRP variance explained by the genetic score and both the methylation and genetic score combined [6]. Additionally, the interaction between the methylation and genetic score on CRP was studied using a multiplicative interaction term. Finally, we assessed the association between the genetic and methylation scores.

### Association with cardiometabolic phenotypes

The association between the significant CpGs and BMI, total cholesterol, HDL-cholesterol, triglycerides, fasting glucose, fasting insulin, prevalent diabetes, prevalent CHD, and incident CHD was explored in CHS, FHS, InCHIANTI, KORA, NAS, RS, and WHI. The analyses on fasting glucose and fasting insulin only included non-diabetic individuals. Diabetes was defined as fasting glucose ≥7.0 mmol/L, non-fasting glucose ≥11.1 mmol/L or the use of glucose-lowering medication. The lipid traits and fasting glucose were analyzed in mmol/L, whilst fasting insulin was analyzed in pmol/L. Fasting insulin and triglycerides were natural log-transformed. CHD (available in ARIC, CHS, EPICOR, FHS, KORA, NAS, RS, and WHI) was defined as fatal or non-fatal myocardial infarction, coronary revascularization, and unstable angina. The statistical models for the cross-phenotype analyses were similar to the basic CRP model (including age, sex, white blood cell counts, technical covariates, and smoking) with DNA methylation as the dependent variable. The associations were also adjusted for BMI, except the association with BMI itself. We conducted fixed effect meta-analyses using the inverse-variance method for total cholesterol, HDL-cholesterol, fasting glucose, fasting insulin, and prevalent diabetes. For incident CHD, associations were analyzed using (penalized) Cox regression models. Results of the cross-phenotype associations with BMI and triglycerides were meta-analyzed combining *P* values, taking into account the study sample size and direction of effect. Both methods are implemented in METAL. We used a Bonferroni corrected *P* value of 0.05 divided by the number of significant CpGs multiplied by nine phenotypes as a threshold of significant cross-phenotype association.

### Gene expression analyses

To assess the relations of replicated CpGs with gene expression, we examined the association between replicated CpGs and whole blood gene expression of *cis*-genes (250 kb upstream and downstream of the CpG). The methylation-expression analyses were conducted in 3699 individuals from the FHS, KORA, and RS with both DNA methylation and gene expression available from the same blood samples. In RS and KORA, we first created residuals for both DNA methylation and messenger RNA (mRNA) expression after regressing out age, sex, blood cell counts (fixed effect), and technical covariates (random effect). We then examined the association between the residuals of DNA methylation (independent variable) and mRNA expression (dependent variable) using a linear regression model. In FHS, we removed 25 surrogate variables (SVs) [26] from the gene expression, along with sex, age, and imputed blood cell fractions as fixed effects, and technical covariates, such as batch effects and lab effects as random effects. We also removed 25 separately computed SVs from the methylation data, along with sex, age, and imputed blood cell fractions as fixed effects, and technical covariates, such as batch effects and lab effects as random effects. We then associated the two data using a simple linear model. Expression probes were aligned to genes and unique methylation-gene expression results from FHS (*n* = 2262), KORA (*n* = 707), and RS (*n* = 730) were meta-analyzed using the sample size weighted method implemented in METAL, based on *P* values and direction of the effects. To reduce the type 1 error, results for the methylation-expression associations were adjusted for multiple testing using the Bonferroni correction (0.05/590 tests: *P* < 8.47 × 10^−5^). Furthermore, for the significant methylation-expression associations, we tested the association between the gene expression and serum CRP levels. We examined the association between gene expression (dependent variable) and CRP levels (independent variable) in a linear model adjusted for age, sex, blood cell counts, technical covariates (plate ID and RNA quality score), tobacco smoking, and BMI. Results from GTP (*n* = 114), FHS (*n* = 5328), InCHIANTI (*n* = 590), KORA (*n* = 724), and RS (*n* = 870) were meta-analyzed using the sample size weighted method implemented in METAL (*P* < 0.05 was considered significant) [24]. Information on gene expression quantification in the specific studies can be found in Additional file 13: Supplemental methods.

### Genetic correlates of DNA methylation

We studied genetic variants in the proximity (±250 kb) of the inflammation-related CpGs for a methylation quantitative trait effect on the percentage of methylation of the CpG site (*cis*-mQTL). The discovery analyses were conducted in the RS in which 730 participants were available with both genetic and epigenetic data. Genotyping information for the RS is described in Additional file 13: Supplemental methods. We used the expression quantitative trait loci (eQTL) mapping pipeline to study associations between genetic variants in a 500 kb window around the CpG site and the percentage of methylation at this CpG site [27]. This pipeline has been applied previously to study eQTL. Instead of analyzing gene expression, we modeled the correlation between genetic variants and DNA methylation and adjusted for 20 principal components derived from the DNA methylation data to account for potential unrelated variation in the DNA methylation caused by environmental or technical effects (batch effects). The threshold of significance for *cis*-mQTLs was defined according to the pipeline specifications by a false discovery rate of 5%. When multiple *cis*-mQTLS were identified for the same CpG site, only the SNP with the lowest *P* value was reported. Next, significant *cis*-mQTLs were replicated in FHS. The *cis*-mQTL analysis in FHS was performed on 2408 individuals having both genotype and methylation data. Genotyping information for FHS is described in Additional file 13: Supplemental methods. We removed 50 principal components from the epigenomics data, along with sex, age, and imputed blood cell fractions as fixed effects, and technical covariates, such as batch effects and lab effects as random effects. We then associate the epigenomic residual data with the genotypic data accounting for ten principal components computed using the Eigenstrat software using fixed effect linear model. We collected effect value, T statistics, and *P* value. We used a Bonferroni corrected *P* value of 0.05/20 = 2.5 × 10^−3^ (based on 20 findings in the discovery) for significant replication in FHS. Subsequently, replicated *cis*-mQTLs were tested for association with serum CRP in the largest published CRP GWAS (*n* = 66,185) to strengthen the causal inference from our findings [6].

### GWAS catalog, pathway analysis, and tissue enrichment

We used the National Human Genome Research Institute (NHGRI) GWAS catalog to query whether genes annotated to replicated CpGs were enriched for genes identified in published GWAS [28]. Altogether, 7600 SNPs, annotated to 4498 genes, associated with 988 phenotypes at GWAS *P* value ≤ 5 × 10^−8^, were retrieved on 25 August 2016 from the NHGRI GWAS catalog. Methylation CpGs were matched by gene symbols with the reported genes in the GWAS catalog. CpGs not annotated to a gene were discarded. Enrichment statistics were performed using one-sided Fisher’s test. Next, enrichment of canonical pathways was explored using Ingenuity® Pathway Analysis software tool (IPA®, QIAGEN Redwood City, http://www.qiagen.com/ingenuity). Replicated CpGs which mapped to a UCSC Refseq gene were included in pathway analyses. Pathway analyses were performed using the IPA software tool (IPA build version 338830 M, content version: 23814503, release date 2016-10-04, analysis date 2015-08-03; http://www.ingenuity.com/). Gene enrichment in canonical pathways was assessed in the core analysis module using Fisher’s exact test right-tailed. Furthermore, we used experimentally derived Functional element Overlap analysis of ReGions from EWAS (eFORGE) to identify tissue specific or cell-type specific signals [29]. eFORGE analyzes a set of differentially methylated CpGs for enrichment of overlap with DNase 1 hypersensitivity sites in different cell types of the ENCODE project. All 58 replicated CpGs were entered as the input of the eFORGE analysis. The set of 58 CpGs were tested for enrichment for overlap with putative functional elements compared to matched background CpGs. The functional elements considered are DNase I hotpsots fromthe ENCODE project. The matched background is a set of the same number of CpGs as the test set, matched for gene relationship and CpG island relationship annotation. Thousand matched background sets were applied. The enrichment analysis was performed for different tissues, since functional elements may differ across tissues. Enrichment outside the 99.9th percentile (−log10 binomial *p* value: ≥3.38) was considered statistically significant (red).

## Additional files

Additional file 1: Table S1. Clinical characteristics of the individuals in the discovery and replication studies. (PDF 590 kb)
Additional file 2: Table S2. Study-specific quality control and analysis details. (PDF 475 kb)
Additional file 3: Table S3. Discovery and replication results of the 218 significant associations in the discovery population. (XLSX 41 kb)
Additional file 4: Table S4. Study-specific effect estimates for the replicated hits. (PDF 330 kb)
Additional file 5: Table S5. Meta-analysis results including all European and African-American whole blood studies. (XLSX 25 kb)
Additional file 6: Figures S1–S4. (PDF 1036 kb)
Additional file 7: Table S6. Association results of the 58 replicated hits in CD4+ cells in the GOLDN study. (XLSX 14 kb)
Additional file 8: Table S7. Associations of the replicated loci with nine cardiometabolic phenotypes. Significant CpG-phenotype associations are highlighted in yellow. (XLSX 12 kb)
Additional file 9: Table S8. Significant methylation-expression results and corresponding expression-CRP results for the replicated CpGs. (XLSX 11 kb)
Additional file 10: Table S9. Significant associations between DNA methylation and corresponding nearby genetic variants and between the genetic variant and CRP in the largest published GWAS of CRP (*n* = 66,185). (XLSX 12 kb)
Additional file 11: Table S10. GWAS enrichment analysis. (PDF 335 kb)
Additional file 12: Table S11. Canonical pathways identified using IPA. (PDF 339 kb)
Additional file 13: Supplemental text. (PDF 1005 kb)

## Abbreviations

BMI: Body mass index
CHD: Coronary heart disease
CpG: Cytosine-phosphate-guanine
CRP: C-reactive protein
DNA: Deoxyribonucleic acid
eQTL: Expression quantitative trait locus
EWAS: Epigenome-wide association study
GWAS: Genome-wide association study
HDL-cholesterol: High-density lipoprotein cholesterol
mQTL: Methylation quantitative trait locus
NHGRI: National Human Genome Research Institute
SNP: Single nucleotide polymorphism
SV: Surrogate variable
