# Supplementary material for: DNA methylation signatures of chronic low-grade inflammation are associated with complex diseases
Source: Genome Biol. 2016 Dec 12;17:255. doi: 10.1186/s13059-016-1119-5 (PMC5151130; doi:10.1186/s13059-016-1119-5)
Supplement: Additional file 1: Table S1. — Clinical characteristics of the individuals in the discovery and replication studies. (PDF 590 kb) [file 13059_2016_1119_MOESM1_ESM.pdf]

Supplementary Table 1. Clinical Characteristics of the individuals in the discovery and replication studies.

	N	Female (%)	Age (years)	CRP (mg/L)	BMI (kg/m^2)	SBP (mmHg)	TC (mmol/L)	FG (mmol/L)	Diabetes (%)	CHD (%)	Smoking (%)	HTN (%)	Lipid Rx (%)
Discovery cohorts													
CHS (EA)	187	56	76 (5)	6.6 (11.0)	27 (5)	136 (22)	5.3 (1.1)	5.9 (1.5)	12	3	13	42	5
EPIC	1287	51	60 (9)	3.3 (5.4)	27 (4)	NA	NA	NA	NA	NA	27	NA	NA
FHS	2427	52	66 (9)	3.1 (6.7)	28 (5)	129 (17)	4.8 (1.0)	5.9 (1.3)	14	7	11	55	44
GOLDN	976	52	49 (16)	2.4 (4.4)	28 (6)	116 (17)	2.0 (0.9)	5.6 (0.9)	8	0	7	35	0
InCHIANTI	498	55	63 (16)	3.2 (3.5)	27 (4)	141 (21)	5.6 (1.0)	5.1 (1.2)	8	7	19	58	9
KORA	1700	51	61 (9)	2.3 (3.7)	28 (5)	125 (19)	5.7 (1.0)	5.6 (1.1)	9	3	14	37	16
LBC 1921	169	54	87 (0)	3.7 (8.4)	26 (4)	168 (25)	NA	NA	6	NA	3	47	NA
LBC 1936	296	50	70 (1)	5.3 (6.8)	28 (4)	149 (19)	5.4 (1.2)	NA	8	NA	11	41	NA
NAS	648	0	73 (7)	3.3 (6.1)	28 (4)	131 (17)	5.0 (1.0)	6.0 (1.5)	19	19	4	71	38
RS	702	54	60 (8)	2.7 (4.7)	28 (5)	134 (14)	5.5 (1.1)	5.6 (1.2)	10	6	27	62	27
WHI EA*	478	100	69 (6)	4.9 (6.4)	29 (6)	129 (19)	6.2 (1.0)	6.1 (2.1)	14	100	1.5	48	11
WHI EA†	471	100	68 (6)	3.8 (5.5)	28 (6)	126 (17)	6.0 (1.0)	5.5 (1.3)	4	0	0.6	27	8
Replication cohorts													
ARIC	2264	64	56 (6)	5.9 (7.8)	30 (6)	126 (20)	5.4 (1.1)	7.2 (3.6)	25	4	25	55	4
CHS (AA)	193	65	73 (5)	5.2 (5.6)	29 (5)	142 (21)	5.5 (0.9)	6.6 (2.7)	24	1	15	59	4
GENOA	939	71	66 (8)	6.7 (12.3)	31 (6)	140 (21)	5.3 (1.1)	6.2 (2.3)	31	7	13	82	21
GTP	112	70	41 (13)	5.9 (8.1)	33 (8)	NA	NA	NA	NA	NA	NA	NA	NA
WHI AA*	294	100	64 (7)	7.2 (8.4)	32 (6)	133 (19)	6.1 (1.3)	6.9 (3.3)	27	100	3.1	64	13
WHI AA†	309	100	62 (6)	6.1 (7.5)	31 (7)	129 (17)	5.8 (1.1)	5.8 (2.0)	10	0	0.3	52	6
