# Supplementary material for: DNA methylation signatures of chronic low-grade inflammation are associated with complex diseases
Source: Genome Biol. 2016 Dec 12;17:255. doi: 10.1186/s13059-016-1119-5 (PMC5151130; doi:10.1186/s13059-016-1119-5)
Supplement: Additional file 2: Table S2. — Study-specific quality control and analysis details. (PDF 475 kb) [file 13059_2016_1119_MOESM2_ESM.pdf]

Supplementary Table 2. Study-specific QC and analysis details.

Study	Platform	Tissue	*QC details* Background correction	N bead filtering	Detection P value	Probe call rate	Sample call rate	Outliers removal	Sex-mismatch	Genotype-mismatch	Normalization	*Analysis details* Study-specific covariate	Batch effect adjustment	Cell composition
ARIC	Illumina HM 450K BeadChip	Whole blood	yes	yes (<3 functional beads)	0.01	99%	95%	yes (PCA)	yes	yes	BMIQ	10 ancestry PC	chip, row, column	Estimated
CHS AA	Illumina HM 450K BeadChip	Whole blood	yes	no	0.01	99%	99.5%	yes	yes	yes	SWAN	5 ancestry PC	chip, row, column	Estimated
CHS EA	Illumina HM 450K BeadChip	Whole blood	yes	no	0.01	99%	99.5%	yes	yes	yes	SWAN	-	chip, row, column	Estimated
EPIC-Norfolk	Illumina HM 450K BeadChip	Whole blood	yes	no	0,01	95%	95%	no	no	no	SWAN	-	chip, row, column	Estimated
FHS	Illumina HM 450K BeadChip	Whole blood	yes	no	0.01	80%	99%	yes (MDS)	yes	yes	DASEN	Pedigree	chip, row, column	Estimated
GENOA	Illumina HM 27K BeadChip	Whole blood	yes	no	0.01	99%	95%	no	no	no	quantile normalization (lumiMethyN)	Pedigree	chip, row, column	Estimated
GOLDN	Illumina HM 450K BeadChip	CD4+ cells	no	yes (<3 functional beads)	0.01	90%	98.5%	no	yes	no	ComBat	Pedigree, 4 T-cell purity PC	plate, position on plate	-
GTP	Illumina HM 450K BeadChip	Whole blood	yes	yes (<3 functional beads)	0.001	95%	95%	yes (HC)	yes	no	Quantile normalization of signal	-	chip, row	Estimated
InCHIANTI	Illumina HM 450K BeadChip	Whole blood	yes	yes (<3 functional beads)	0.01	95%	95%	yes	yes	yes	DASEN	Study site	plate	Estimated
KORA	Illumina HM 450K BeadChip	Whole blood	yes (lumi)	yes (<3 functional beads)	0.01	95%	80%	no	no	no	BMIQ	-	chip, row, column	Estimated
LBC1921	Illumina HM 450K BeadChip	Whole blood	yes	no	0.01	95%	93%	no	yes	yes	-	-	plate, chip, position on chip, hybridisation date	Measured
LBC1936	Illumina HM 450K BeadChip	Whole blood	yes	no	0.01	95%	93%	no	yes	yes	-	-	plate, BeadChip, position on BeadChip,	Measured
NAS	Illumina HM 450K BeadChip	Whole blood	yes	yes (<3 functional beads)	0.05	95%	95%	no	yes	yes	BMIQ	-	plate, chip, row, column	Measured
Rotteram	Illumina HM 450K BeadChip	Whole blood	yes	no	0.01	99%	99%	no	yes	no	DASEN	-	chip, row, column	Measured
WHI AA cases	Illumina HM 450K BeadChip	Whole blood	no	yes (<3 functional beads)	0.01	90%	98.5%	no	yes	no	ComBat	-	chip	Estimated
WHI AA controls	Illumina HM 450K BeadChip	Whole blood	no	yes (<3 functional beads)	0.01	90%	98.5%	no	yes	no	ComBat	-	chip	Estimated
WHI EA cases	Illumina HM 450K BeadChip	Whole blood	no	yes (<3 functional beads)	0.01	90%	98.5%	no	yes	no	ComBat	-	chip	Estimated
WHI EA controls	Illumina HM 450K BeadChip	Whole blood	no	yes (<3 functional beads)	0.01	90%	98.5%	no	yes	no	ComBat	-	chip	Estimated
