# Supplementary material for: DNA methylation signatures of chronic low-grade inflammation are associated with complex diseases
Source: Genome Biol. 2016 Dec 12;17:255. doi: 10.1186/s13059-016-1119-5 (PMC5151130; doi:10.1186/s13059-016-1119-5)
Supplement: Additional file 4: Table S4. — Study-specific effect estimates for the replicated hits. (PDF 330 kb) [file 13059_2016_1119_MOESM4_ESM.pdf]

MarkerName	CHS EA	EPIC	FHS	InCHIANTI	KORA	LBC1921	LBC1936	NAS	RS	WHI case EA	WHI control EA	ARIC	CHS AA	GENOA	GTP	WHI case AA	WHI control AA
**cg00159243**	-0,0026	-0,0021	-0,00295	-0,00603	-0,00154	-0,00364	-0,00381	-0,0041	-0,00261	-0,00381	-0,00231	-0,00415	-0,00337	-	-0,00375	-0,00554	-0,00083
**cg00812761**	0,0051	0,00018	0,00192	-0,00031	0,00326	-0,00162	0,00015	0,0024	0,00552	0,00378	0,0024	0,00401	0,00496	-	0,00364	0,004	0,00136
**cg00851028**	0,00536	0,00153	0,00207	0,0018	0,00219	0,00421	0,00311	0,00209	0,00305	0,00613	0,00158	0,00478	0,00361	-	0,00371	0,0033	0,00426
**cg01059398**	-0,0084	-0,00486	-0,00391	-0,00106	-0,00593	-0,00216	0,00057	-0,00583	-0,00403	-0,00036	-0,00367	-0,00601	-0,00763	-	-0,00272	-0,01095	-0,00656
**cg01409343**	-0,003	-0,00515	-0,00426	-0,00246	-0,00414	-0,00635	0,00223	-0,01063	-0,00091	-0,00084	-0,00287	-0,01151	-0,00377	-	-0,00101	-0,00697	-0,00534
**cg02003183**	0,00645	0,00256	0,00518	3,00E-05	0,00303	0,01044	0,00863	0,00537	0,00471	0,00771	0,00627	0,00675	0,0055	-	0,00098	0,00333	0,00319
**cg02341197**	0,00532	0,0044	0,00285	-0,00197	0,00417	-6,00E-04	-0,00562	0,00488	0,00138	0,00614	0,00513	0,00521	0,00086	-	0,00561	0,00829	0,00027
**cg02481950**	0,0034	0,00194	0,00238	0,00201	0,00223	0,00191	0,00187	0,0049	0,001	0,0038	0,00285	0,00347	0,00129	-	0,00351	0,00449	0,00413
**cg02650017**	-0,0044	-0,00405	-0,00216	-0,00243	-0,00152	-0,00095	-0,00228	-0,00188	-0,00306	-0,00173	-0,0027	-0,00108	0,00139	-	-0,00294	-0,00181	-0,00112
**cg02734358**	-0,00477	-0,00561	-0,00381	-0,00509	-0,00616	-0,01287	0,00536	-0,00394	-0,00642	-0,00249	-0,00813	-0,00673	-0,00089	-	-0,00087	-0,00696	-0,00492
**cg03128029**	-0,00067	-0,00302	-0,00242	-0,00311	-0,00241	-0,00283	-0,00066	-0,00869	-0,00218	-0,00169	-0,00564	-0,00617	-0,00404	-	0,00032	-0,00191	-0,00097
**cg03957124**	-0,00359	-0,00195	-0,00265	-0,00161	-0,00485	0,00156	-0,00235	-0,0042	-0,00268	-0,00069	-0,00452	-0,00499	-0,00541	-	-0,00098	-0,00054	-0,00105
**cg04523589**	0,00256	0,00034	0,00262	0,00154	0,0024	0,00283	0,00287	0,0032	8,00E-04	0,00449	0,0038	0,00305	0,00558	0,00174	0,00676	0,00505	0,00111
**cg04987734**	0,00724	-1,00E-04	0,00522	-0,001	0,00375	0,0068	0,00183	0,00605	0,0029	0,00875	0,00447	0,00775	0,00646	-	-0,00073	0,00516	0,00092
**cg05316065**	-0,00188	-0,00405	-0,00196	-0,00352	-0,00429	-0,00178	-0,0043	-0,00389	-0,00222	-0,00057	-0,00404	-0,00576	0,00102	-0,00687	-0,00469	-0,00235	-0,00509
**cg05575921**	0,00224	-0,00687	-0,00496	-0,01077	-0,0072	-0,0061	-0,00739	-0,004	-0,0095	-0,00571	-0,00369	-0,00866	-0,00302	-	-0,0036	-4,00E-05	-0,00726
**cg06126421**	-0,00682	-0,00605	-0,00488	-0,00608	-0,00749	-0,00219	-0,00989	-0,00211	-0,00379	-0,00439	-0,00596	-0,00782	9,00E-05	-	0,00064	-0,00537	-0,0092
**cg06192883**	0,00626	0,00489	0,00471	0,00218	0,00377	0,00778	0,00482	0,00434	0,00332	0,00601	0,00662	0,00766	0,00649	-	0,00393	0,00881	0,00712
**cg06690548**	0,00211	-0,00122	-0,01023	-0,00201	-0,00626	0,00012	-0,00495	-0,0131	-0,00249	-0,00689	-0,0077	-0,00405	-0,00283	-0,00251	-0,00228	-0,00099	-0,00301
**cg07094298**	-0,00343	-0,00637	-0,00521	-0,00221	-0,00808	-0,00895	-0,00033	-0,00239	-0,00611	-0,00672	-0,00431	-0,00682	-0,00624	-	0,00169	-0,00871	-0,0036
**cg07573872**	-0,00941	-0,00306	-0,00539	-0,00373	-0,00521	-0,00201	-0,00732	-0,01102	-0,00423	-0,00451	-0,00537	-0,00863	-0,00591	-	0,00685	-0,00896	-0,00637
**cg08548559**	-0,00346	-0,00116	-0,00322	-0,00551	-0,00523	-0,00154	0,00074	-0,0036	-0,00429	-0,0057	-0,00675	-0,00552	-0,00335	-	-0,0023	-0,00592	-0,00449
**cg09182678**	-0,00245	-0,00288	-0,00143	-0,00386	-	-0,00349	-0,00787	-0,00042	-0,00482	-0,00065	-0,00238	-0,0023	0,00041	-	-0,0023	-0,00174	-0,00191
**cg10636246**	-0,00246	-0,00672	-0,00739	-0,00516	-0,00693	-3,00E-04	-0,00568	-0,01065	-0,00608	-0,00649	-0,00781	-0,0115	0,00104	-	-0,00336	-0,00722	-0,00923
**cg12053291**	0,00113	0,0019	0,0039	-2,00E-05	0,00315	0,00134	0,00599	0,00476	0,00186	0,00386	0,0016	0,00451	-3,00E-05	-	0,00322	0,00245	0,00487
**cg12054453**	-0,0051	-0,00483	-0,00855	-0,00952	-0,01013	0,00072	-0,01254	-0,01425	-0,00654	-0,00141	-0,01008	-0,01584	-0,00755	-	0,00193	-0,01223	-0,00923
**cg12269535**	-0,00239	-0,00456	-0,00207	-0,00374	-0,00378	-0,0102	-0,00052	-0,00168	-0,00263	-0,00091	-0,00346	-0,00518	-0,00502	-	0,00235	-0,00454	-0,00675
**cg12992827**	-0,00353	-0,00228	-0,00844	-0,00769	-0,00661	-0,01053	0,00311	-0,01077	-0,0015	-0,00455	-0,00856	-0,01055	-0,00112	-	-0,00393	-0,01113	-0,00886
**cg13585930**	-0,0031	-0,00421	-0,00254	-0,00594	-0,00436	-0,00411	-0,00646	-0,00382	-0,00468	-0,00657	-0,00607	-0,0064	-0,0025	-	-0,00085	-0,00393	-0,00573
**cg15020801**	0,00525	0,00322	0,00221	0,00288	0,00115	0,00486	0,00498	0,00534	0,00094	0,00288	0,00478	0,00377	0,00559	-	-8,00E-05	0,00242	0,00324
**cg15310871**	0,00238	0,00323	0,00338	0,00226	0,00106	0,00715	0,00135	0,00207	0,00065	0,00175	0,00179	0,00344	0,00351	-	0,0017	0,00132	0,00169
**cg15551881**	0,00253	0,00195	0,00378	-0,00055	0,00486	0,00553	0,01334	0,00734	0,00194	0,00407	0,00458	0,00571	0,00614	0,00412	0,00968	0,00616	0,00215
**cg15721584**	0,00013	0,00942	0,00493	0,00091	-	-	-	0,00853	0,00383	0,0083	0,01541	0,00626	0,01305	-	0,00106	0,00718	0,01032
**cg16936953**	-0,00759	-0,00537	-0,00846	-0,0073	-0,00978	-0,00542	-0,00631	-0,0152	-0,00467	-0,00243	-0,00849	-0,01681	-0,00837	-	0,00044	-0,01128	-0,00963
**cg17501210**	-0,00572	-0,00599	-0,00722	-0,00297	-0,00838	-0,00697	-0,00184	-0,00614	-0,00551	-0,00529	-0,01064	-0,00648	-0,00231	-	-0,00456	-0,01355	-0,0092
**cg17980786**	0,00531	0,00188	0,00228	4,00E-04	0,00272	0,00445	0,00171	0,00138	0,00339	0,00361	0,00516	0,00603	0,00531	-	0,00591	0,00467	0,00438
**cg18181703**	-0,00755	-0,00426	-0,00522	-0,00359	-0,00651	-0,00722	-0,00425	-0,00543	-0,00518	-0,00398	-0,00301	-0,01212	-0,01103	-	-0,00324	-0,00401	-0,00653
**cg18608055**	-0,00162	-0,00528	-0,00465	-0,00085	-0,00479	-0,00542	0,00058	-0,00398	-0,00419	-0,00244	-0,00473	-0,01017	-0,00579	-	-0,00046	-0,00727	-0,00703
**cg18663307**	0,00023	0,00162	0,0035	0,00236	0,00329	0,00358	0,00367	0,00511	0,00204	0,00169	0,00223	0,00374	0,00519	-	0,00554	0,00491	0,00629
**cg18942579**	-0,00346	-0,00362	-0,00625	-0,00635	-0,00806	-0,00689	0,00695	-0,00983	-0,00438	-0,00218	-0,00432	-0,01567	-0,0057	-	-0,00122	-0,00862	-0,00386
**cg19769147**	-0,00055	0,00263	0,00302	7,00E-04	0,00262	0,00371	0,00548	0,00239	0,00281	0,00461	0,00364	0,0034	-0,00036	-	0,00362	0,00222	0,00229
**cg19821297**	-0,00452	-0,00601	-0,0046	-0,00471	-0,00738	-0,00541	0,00267	-0,00718	-0,00455	-0,00353	-0,00562	-0,00767	-0,00062	-	0,00329	-0,00892	-0,00375
**cg20995564**	-0,00567	-0,00251	-0,00549	-0,00639	-0,0061	-0,0073	-0,00154	-0,00871	-0,00247	-0,00469	-0,00733	-0,01198	-0,00355	-	-0,00106	-0,0073	-0,0103
**cg21429551**	0,00155	-0,00165	-0,0058	-0,00917	-	-0,0043	-0,00451	-0,00715	-0,00853	-0,01002	-0,01789	-0,00972	-0,00632	-	0,00059	-0,00601	-0,01085
**cg22749855**	-0,00302	-0,00277	-0,00284	-0,00064	-0,00286	0,00098	-0,00179	-0,004	-0,00078	-0,00126	-0,00346	-0,00419	-0,00297	-	-0,00177	-0,00305	-0,00407
**cg23761815**	0,00138	0,00261	0,00232	0,00116	0,00279	0,00414	0,00044	0,00209	0,00159	0,00567	0,00121	0,0026	0,00448	-	0,00244	0,00515	0,00156
**cg24174557**	-0,00411	-0,00195	-0,00298	-0,00583	-	-	-	-0,00509	-0,00488	-0,00563	-0,00582	-0,00685	-0,00278	-	8,00E-04	-0,00732	-0,00271
**cg25325512**	-0,00645	-0,00383	-0,00311	-8,00E-04	-0,00541	-0,00137	-0,00567	-0,00551	-0,00127	0,00217	0,00108	-0,0069	-0,00583	-	-0,00458	-0,00124	-0,0045
**cg25392060**	0,00624	0,00481	0,00226	-0,00236	0,00201	0,00409	0,0012	0,00157	0,0029	0,00483	0,00428	0,00349	0,00252	-	0,00389	0,00589	0,00193
**cg26470501**	0,00078	-0,00285	-0,00464	-0,00243	-0,00634	-0,00634	-0,00781	-0,00394	-0,00394	-0,00295	-0,00426	-0,00671	-0,00479	-	0,00094	-0,00466	-0,00461
**cg26610247**	0,00263	0,00366	0,0033	-0,0026	0,00193	0,00774	-0,00029	0,00168	0,00479	0,00232	0,00491	0,00396	0,00576	-	0,00688	0,00392	0,00234
**cg26804423**	0,00393	0,00197	0,00364	0,00056	0,00365	0,00396	-0,00076	0,00187	0,00097	0,00453	0,00156	0,00397	0,00396	-	0,0057	0,00369	0,00109
**cg26846781**	0,00119	0,00199	0,00181	0,00138	0,00183	-0,00192	0,002	0,00385	0,00148	0,00433	0,00162	0,0036	3,00E-05	-	0,00499	0,00376	0,00248
**cg27023597**	-0,00667	-0,00713	-0,0052	-0,00671	-	-	-	-0,00974	-0,00208	-0,00284	-0,0025	-0,01062	-0,00433	-	0,00226	-0,00936	-0,00439
**cg27050612**	0,00014	-0,00267	-0,00183	-0,00204	-0,00177	-0,0054	-0,0034	-0,00054	-0,00269	-0,00154	-0,00135	-0,00308	-0,00318	-	-0,002	-0,00254	-0,0031
**cg27184903**	0,00237	0,00172	0,00311	0,00104	0,00166	0,00125	0,00283	0,00024	-	0,00728	0,00215	0,00389	0,00654	-	0,00448	0,00823	0,00665
**cg27469606**	-0,00191	-0,00161	-0,002	-0,00234	-0,00361	-0,00219	-0,00365	-0,00345	-0,00018	0,00067	-0,00274	-0,0022	-0,00173	-	0,00026	-0,00346	-0,00368
**cg27637521**	-0,00383	-0,00072	-0,00173	0,00065	-0,00078	-0,0062	-0,00564	-0,00137	-0,00108	-0,00164	-0,00228	-0,00165	-0,00047	-0,00383	-0,00142	-0,00114	-0,00058
