# Supplementary material for: DNA methylation signatures of chronic low-grade inflammation are associated with complex diseases
Source: Genome Biol. 2016 Dec 12;17:255. doi: 10.1186/s13059-016-1119-5 (PMC5151130; doi:10.1186/s13059-016-1119-5)
Supplement: Additional file 6: Figures S1–S4. — (PDF 1036 kb) [file 13059_2016_1119_MOESM6_ESM.pdf]

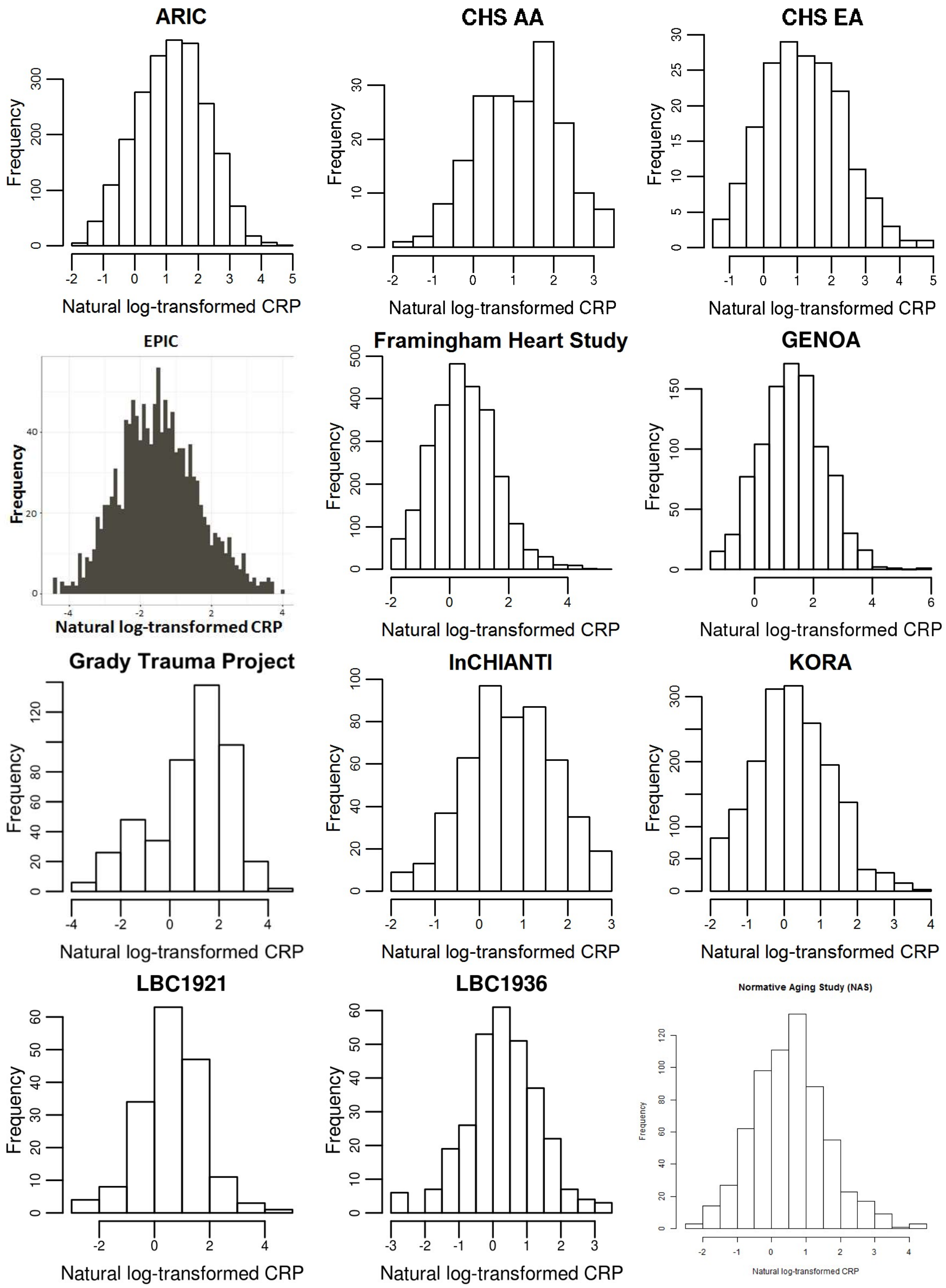
ARIC
Frequency
Natural log-transformed CRP
CHS AA
Frequency
Natural log-transformed CRP
CHS EA
Frequency
Natural log-transformed CRP
EPIC
Frequency
Natural log-transformed CRP
Framingham Heart Study
Frequency
Natural log-transformed CRP
GENOA
Frequency
Natural log-transformed CRP
Grady Trauma Project
Frequency
Natural log-transformed CRP
InCHIANTI
Frequency
Natural log-transformed CRP
KORA
Frequency
Natural log-transformed CRP
LBC1921
Frequency
Natural log-transformed CRP
LBC1936
Frequency
Natural log-transformed CRP
Normative Aging Study (NAS)
Frequency
Natural log-transformed CRP

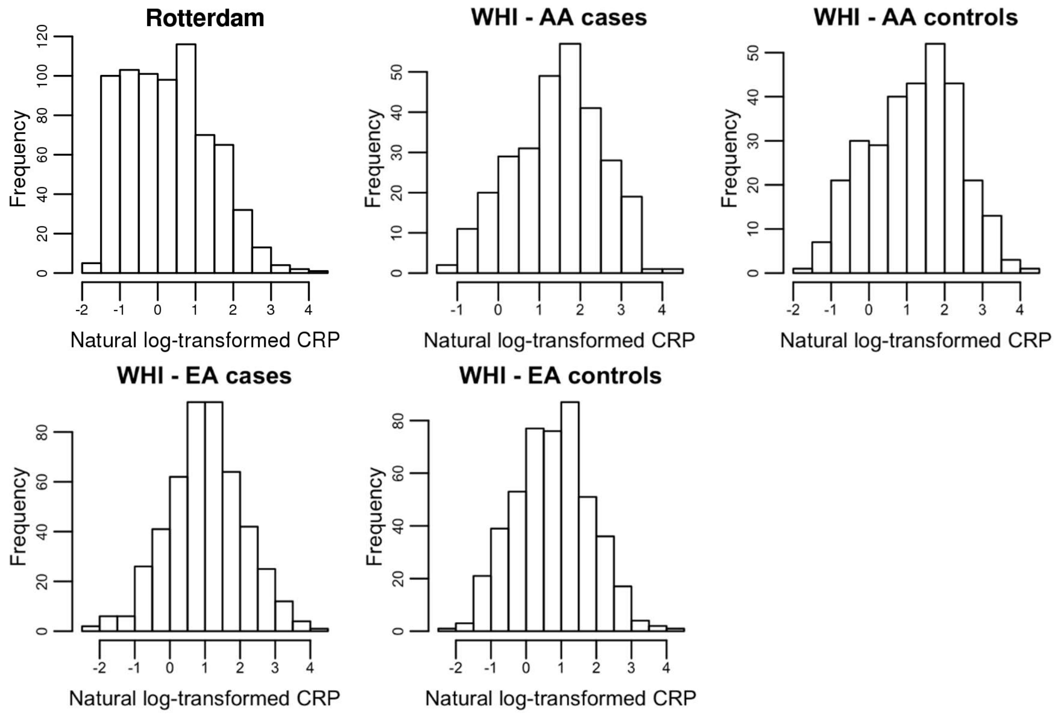

Figure S1. Distributions of the natural log-transformed CRP values in the participating studies.

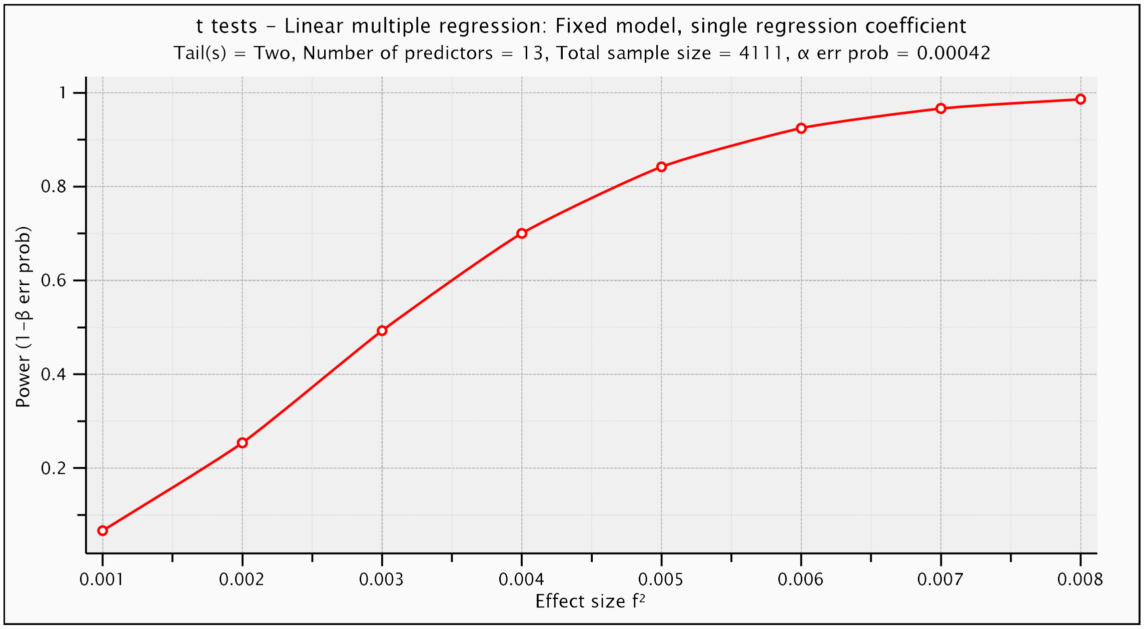

Figure S2. Power calculation for the replication study in African-Americans. The red line depicts the power in the replication study of 4,111 African-Americans to detect the effect size of the association between DNA methylation and CRP obtained in the discovery study presented on the x-axis.

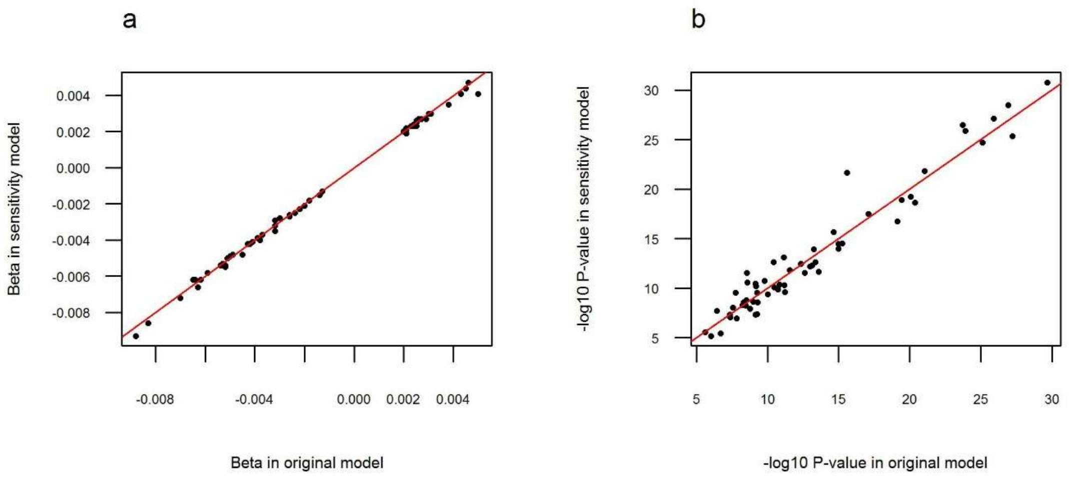

Figure S3. Correlation plots of the effect estimates and –log10 P-values between original model and sensitivity model. a, Correlation plot of the effect estimates (Beta) in original model (adjusted for age, sex, batch covariates, BMI, smoking, and cell counts) and the sensitivity model (additionally adjusted for waist circumference, total/ HDL-cholesterol ratio, prevalent diabetes, hypertension treatment, lipid treatment, hormone replacement therapy, and prevalent coronary heart disease). The correlation between the effect estimates was r=0.999. **b**, Correlation plot of the –log10 P-values between the original and the sensitivity model. The correlation between the –log10 P-values was r=0.973.

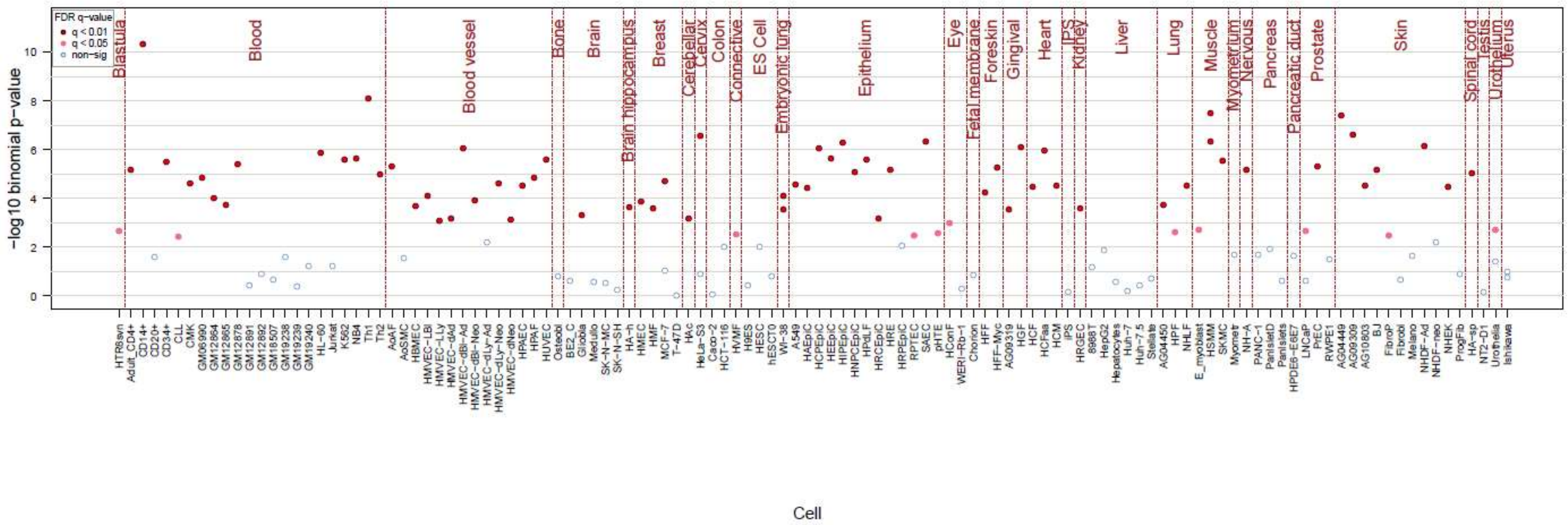

Figure S4. Tissue-specific enrichment of DNase 1 hypersensitivity sites in the ENCODE project. We used experimentally-derived Functional element Overlap analysis of ReGions from EWAS (eFORGE) to identify tissue specific or cell-type specific signals. eFORGE analyzes a set of differentially methylated CpG sites for enrichment of overlap with DNase 1 hypersensitivity sites in different cell types of the ENCODE project. Enrichment outside the 99.9th percentile (-log10 binomial p-value: ≥3.38) was considered statistically significant (red).
