# Supplementary material for: DNA methylation signatures of chronic low-grade inflammation are associated with complex diseases
Source: Genome Biol. 2016 Dec 12;17:255. doi: 10.1186/s13059-016-1119-5 (PMC5151130; doi:10.1186/s13059-016-1119-5)
Supplement: Additional file 11: Table S10. — GWAS enrichment analysis. (PDF 335 kb) [file 13059_2016_1119_MOESM11_ESM.pdf]

Supplementary Table 9. Genome-wide association studies enrichment analysis.

Disease	N genes in GWAS	N genes in EWAS	Enrichment P-value	Gene
Epilepsy (generalized)	9	3	6,00E-07	NFE2L1;PNPO;ZEB2
Renal cell carcinoma	7	2	7,97E-05	SCARB1;ZEB2
Lipoprotein-associated phospholipase A2 activity and mass	13	2	2,94E-04	SCARB1;TMEM49
Vitamin E levels	5	1	9,85E-03	SCARB1
Venous thromboembolism (SNP x SNP interaction)	11	1	0,02	TRIM71
Orofacial clefts	13	1	0,03	GSDMC
Celiac disease or Rheumatoid arthritis	14	1	0,03	TRAF1
Intraocular pressure	15	1	0,03	ICA1
Alzheimer's disease	20	1	0,04	BCL3
Alzheimer's disease (late onset)	38	1	0,07	BCL3
Vitiligo	54	1	0,10	SLC29A3
Inflammatory bowel disease	341	2	0,15	RPS6KA2;SBNO2
Rheumatoid arthritis	134	1	0,23	TRAF1
HDL cholesterol	155	1	0,27	SCARB1
Prostate cancer	279	1	0,43	PHOSPHO1
Crohn's disease	300	1	0,45	SBNO2
Height	391	1	0,54	GSDMC
Schizophrenia	405	1	0,56	ZEB2
