# Supplementary material for: DNA methylation signatures of chronic low-grade inflammation are associated with complex diseases
Source: Genome Biol. 2016 Dec 12;17:255. doi: 10.1186/s13059-016-1119-5 (PMC5151130; doi:10.1186/s13059-016-1119-5)
Supplement: Additional file 12: Table S11. — Canonical pathways identified using IPA. (PDF 339 kb) [file 13059_2016_1119_MOESM12_ESM.pdf]

Supplementary Table 10. Enrichment in caninocial pathways using Ingenuity Pathway Analysis.

**Ingenuity Canonical Pathways**	**Molecules**	**p-value**
Growth Hormone Signaling	SRF,SOCS3,RPS6KA2	0.001
IL-9 Signaling	SOCS3,BCL3	0.005
MSP-RON Signaling Pathway	RPS6KA2,ITGB2	0.009
Pyridoxal 5'-phosphate Salvage Pathway	PIM1,PNPO	0.01
STAT3 Pathway	SOCS3,PIM1	0.01
TR/RXR Activation	BCL3,SCARB1	0.02
Type I Diabetes Mellitus Signaling	SOCS3,ICA1	0.03
IGF-1 Signaling	SRF,SOCS3	0.03
Glutaryl-CoA Degradation	GCDH	0.03
p38 MAPK Signaling	SRF,RPS6KA2	0.03
Atherosclerosis Signaling	SELPLG,ITGB2	0.04
IL-6 Signaling	SRF,SOCS3	0.04
phagosome maturation	ATP6V1B2,TUBB	0.04
Tryptophan Degradation III (Eukaryotic)	GCDH	0.05
Inflammasome pathway	AIM2	0.05
