# Supplementary material for: DNA methylation signatures of chronic low-grade inflammation are associated with complex diseases
Source: Genome Biol. 2016 Dec 12;17:255. doi: 10.1186/s13059-016-1119-5 (PMC5151130; doi:10.1186/s13059-016-1119-5)
Supplement: Additional file 13: — Supplemental text. (PDF 1005 kb) [file 13059_2016_1119_MOESM13_ESM.pdf]

Supplementary Text

DNA methylation signatures of chronic low-grade inflammation are associated with complex diseases

Table of Content

a) Study-specific methods section.

b) Genotyping information Framingham Heart Study and Rotterdam Study.

c) Gene expression quantification details.

a. Study-specific methods section

The Atherosclerosis Risk in Communities (ARIC) Study

The Atherosclerosis Risk in Communities (ARIC) Study is a prospective cohort study of cardiovascular disease risk in four U.S. communities. Between 1987 and 1989, 7,082 men and 8,710 women aged 45–64 years were recruited from Forsyth County, North Carolina; Jackson, Mississippi (African Americans only); suburban Minneapolis, Minnesota; and Washington County, Maryland. The ARIC Study protocol was approved by the institutional review board of each participating university. After written informed consent was obtained, including that for genetic studies, participants underwent a baseline clinical examination (Visit 1) and four subsequent follow-up clinical exams (Visits 2 – 5).

At this time, DNA methylation data are available for African American members of the cohort from two centers (Forsyth County and Jackson). The present study comprises a cross-sectional analysis of CRP and methylation measured in samples collected at visit 2, with covariates obtained at the same visit.

Genomic DNA was extracted from peripheral blood leukocyte samples using the Gentra Puregene Blood Kit (Qiagen; Valencia, CA, USA) according to the manufacturer's instructions (www.qiagen.com). Bisulfite conversion of 1 ug genomic DNA was performed using the EZ-96 DNA Methylation Kit (Deep Well Format) (Zymo Research; Irvine, CA, USA) according to the manufacturer's instructions (www.zymoresearch.com). Bisulfite conversion efficiency was determined by PCR amplification of the converted DNA before proceeding with methylation analyses on the Illumina platform using Zymo Research's Universal Methylated Human DNA Standard and Control Primers.

Bisulfite-converted DNA was used for hybridization on the HM450 BeadChip, following the Illumina Infinium HD Methylation protocol (www.illumina.com). This consisted of a whole genome amplification step followed by enzymatic end-point

fragmentation, precipitation and re-suspension. The re-suspended samples were hybridized to the complete set of bead-bound probes, followed by ligation and single-base extension during which a fluorescently-labeled nucleotide is incorporated, and scanned. The degree of methylation is determined for each CpG cytosine by measuring the amount of incorporated label for each probe. The intensities of the images were extracted using Illumina GenomeStudio 2011.1, Methylation module 1.9.0 software. The methylation score for each CpG sites was represented as a beta (β) value according to the fluorescent intensity ratio. Background subtraction was conducted with the GenomeStudio software using built-in negative control bead types on the array.

Positive and negative controls and sample replicates were included on each 96-well plate assayed. After exclusion of controls, replicates, and 22 samples that failed bisulfite conversion, a total of 2,950 study participants had HM450 data available for further quality control analyses. We removed poor-quality samples with pass rate <95%, gender mismatch, SNP discordance with previous genotyping, and outliers in principal component analysis (N = 107). At the target level, we flagged poor-quality CpG sites with average detection p-value > 0.01, and calculated the percentage of samples having detection p-value > 0.01 for each autosomal and X chromosome CpG site. There were 5,174 autosomal and X chromosomal markers where >1% of samples showed detection p-value > 0.01, and these sites were excluded.

Methylation values were normalized using the Beta MIxture Quantile dilation (BMIQ) method.[1] Since white blood cell proportions were not directly measured in most participants in ARIC, they were imputed from the methylation data using the Houseman method.[2] Specifically, the proportions of neutrophils, lymphocytes, monocytes, eosinophils, and basophils were estimated based on the measured differential cell counts available for a subset of ARIC participants at Visit 2 (n = 175). All association analyses were performed in R using

linear mixed models with DNA methylation beta values as the outcome. Analyses were adjusted for age, sex, BMI, smoking status, the top 10 genetic principal components, and estimated white blood cell proportions as fixed effects. Additionally chip, chip row, and column were adjusted for as random effects. Due to availability of CRP measurements, we restricted our analysis to 2,264 samples with both DNA methylation and phenotype data available.

CRP was measured in serum stored at −70°C using an immunoturbidimetric assay on the Roche Modular P chemistry analyzer (Roche Diagnostics, Indianapolis, IN). The coefficient of variation, after excluding outliers, was 7.0%.

Cardiovascular Health Study (CHS)

The CHS is a population-based cohort study of risk factors for coronary heart disease and stroke in adults ≥65 years conducted across four field centers.[3] The original predominantly European ancestry cohort of 5,201 persons was recruited in 1989-1990 from random samples of the Medicare eligibility lists; subsequently, an additional predominantly African-American cohort of 687 persons were enrolled for a total sample of 5,888. CHS was approved by institutional review committees at each field center and individuals in the present analysis had available DNA and gave informed consent including consent to use of genetic information for the study of cardiovascular disease.

DNA methylation was measured on a randomly selected subset of 200 African-Americans participants and 200 European descent participants from study year. Participants from those without presence of coronary heart disease, congestive heart failure, peripheral vascular disease, valvular heart disease, stroke or transient ischemic attack at study baseline or lack of available DNA at study year 5.

Methylation measurements were performed at the Institute for Translational Genomics and Population Sciences at the Harbor-UCLA Medical Center Institute for Translational Genomics and Population Sciences using the Infinium HumanMethylation450 BeadChip (Illumina Inc, San Diego, CA). Quality control was performed in in the minfi R package.[4-6] (version 1.12.0, http://www.bioconductor.org/packages/release/bioc/html/minfi.html). Samples with low median intensities of below 10.5 (log_2) across the methylated and unmethylated channels, samples with a proportion of probes falling detection of greater than 0.5%, samples with QC probes falling greater than 3 standard deviation from the mean, sex-check mismatches, or failed concordance with prior genotyping were removed. In total, 11 samples were removed for sample QC resulting in a sample of 191 European-ancestry and 198 African-American samples. Methylation values were normalized using the SWAN quantile normalization method.[5] Since white blood cell proportions were not directly measured in CHS they were estimated from the methylation data using the Houseman method.[2] All association analyses were performed in R using linear mixed models with DNA methylation beta values as the outcome. Analyses were stratified by race and all analyses were adjusted for age, gender, bmi, current smoking status and estimated white blood cell proportions as fixed effects. Additionally chip, chip row and column were adjusted for as random effects. African-American analyses were also adjusted for five genetic principal components.

CRP was measured from stored serum samples by high-sensitivity ELISA assay (R&D Systems, Minneapolis, MN, USA).[7]

European Prospective Investigation into Cancer and Nutrition (EPIC)

The European Prospective Investigation of Cancer (EPIC)-Norfolk study enrolled more than 25,000 community-based men and women at baseline (1993-1997), who were aged 40-79

years old and registered with a participating general practitioner in and around the city of Norwich (Norfolk, UK). The full details of the study design and follow up of participants has been reported previously.[8] Written informed consent was obtained from all participants. The study complies with the principles of the Declaration of Helsinki and ethical approval was given by the Norfolk Local Research Ethics Committee and the East Norfolk and Waveney NHS Research Governance Committee. Blood samples used for measurement of CRP at baseline were centrifuged at 2,100 g for 15 min at 4 °C and then kept frozen in −80 °C freezers until being thawed in 2008 for assaying CRP. Serum high-sensitivity CRP (mg/l) was measured using the Olympus AU640 chemistry analyzer (Olympus Diagnostics, United Kingdom). In total, 1,287 individuals with DNA methylation, CRP levels and covariate information available were included in the analysis.

EPICOR

EPICOR is a case-control study nested within the European Prospective Investigation into Cancer and Nutrition (EPIC)-Italy cohort.[9] Aim of EPICOR is to investigate dietary- and lifestyle-related cardiovascular risk factors in more than 1,500 subjects form 4 Italian recruitment centers: Turin and Varese (Northern Italy), Naples (Central-Southern Italy), and Ragusa (Southern Italy). All EPICOR cases developed MI after recruitment were identified at cohort follow-up from hospital discharge databases. Healthy controls from the same cohort were matched with cases at recruitment for sex, center and season of recruitment.

All subjects signed an informed consent at their inclusion into the EPIC-Italy cohort. The EPICOR study was approved by the Ethical Committee of the Human Genetics Foundation (Turin, Italy).

A DNA methylation study has been performed on 503 subjects out of the 584 investigated in the context of a case-control subproject on myocardial infarction as described previously[10]

after exclusion of 67 subjects with missing data on hs-CRP and 14 subjects with hs-CRP > 10 mg/L.

DNA was extracted from buffy coat stored in liquid nitrogen, bisulfite converted (EZ-96 DNA Methylation-Gold Kit, Zymo Research Corporation) and methylation levels assessed with the Infinium HumanMethylation450 BeadChip (Illumina Inc., San Diego, CA) according to manufacturer's instructions. Averagemethylation "Beta-values", were computed with the dedicated GenomeStudio Methylation software (v2011.1, llumina Inc., San Diego, CA) as the ratio of the intensity of the methylated signal over the total signal (unmethylated + methylated). Raw data were quality controlled as follows: single Beta-values with detection p-value ≥ 0.01 were excluded from the analysis, as well as (1) CpG loci with detection p-value ≥ 0.01 in more than 20% of the assayed samples, (2) probes containing SNPs with MAF ≥ 0.05 in the CEPH (Utah residents with ancestry from northern and western Europe, CEU) population, and (3) samples with a global call rate ≤ 95%. Further 9,959 CpGs whose methylation signal was detected by cross-hybridizing and SNPs-containing probes were removed, leaving 425,498 CpG sites available for the analyses. Background normalization was performed on raw methylation data according to Marabita et al.[11]

The percentage of WBCs subtypes was estimated from genome-wide methylation data according to Houseman[2] et al and included as correction into the analyses.

Framingham Heart Study (FHS)

The Framingham Offspring studied was initiated in 1971,[12] and was constituted of the children and the children's spouses of the Original cohort.[13] The eligible sample for this investigation was from the 3,021 participants in the FHS offspring cohort who attended the eighth examination cycle from 2005-2008, had peripheral blood samples obtained, high sensitivity C-reactive protein (hsCRP) measures, and gave consent for genomic studies. DNA

methylation, assayed with the Infinium HumanMethylation450 BeadChip [20] (Illumina Inc., San Diego, CA), was available on 2,846 FHS participants. High-sensitivity C-reactive protein was measured on fasting serum samples Dade Behring BN100 nephelometer (Deerfield, Ill). The intra-assay coefficient of variation was 3.2%.[14] Buffy coat preparations were obtained from the whole blood samples. Genomic DNA was extracted using the Gentra Puregene DNA extraction kit (Qiagen, Venlo, Netherlands) which subsequently underwent bisulfite conversion using the EZ DNA Methylation kit (Zymo Research, Irvine, CA). Samples underwent whole genome amplification, fragmentation, array hybridization, and single-base pair extension. DNA methylation results underwent normalization within laboratory batches using the DASEN methodology implemented in the *wateRmelon* package,[15] which includes background adjustment of the methylated and unmethylated intensities and quantile normalization of the methylated and unmethylated probes within the two types of probe technologies separately. We excluded samples with a missing rate >1% at p<0.01, poor single nucleotide polymorphism (SNP) matching to the 65 SNP control probe locations, and outliers by multi-dimensional scaling techniques. We also excluded probes with a missing rate >20% at p<0.01. After quality control exclusions and overlap with individuals with hsCRP measures, there were 2,427 FHS participants available to contribute to analyses. The FHS DNA methylation data is available on dbGAP (accession number: phs000724.v5.p10).

The Genetic Epidemiology Network of Arteriopathy (GENOA)

The Genetic Epidemiology Network of Arteiopathy (GENOA) study is a community-based study of hypertensive sibships that was designed to investigate the genetics of hypertension and target organ damage in African Americans from Jackson, Mississippi and non-Hispanic whites from Rochester, Minnesota.[16] In the initial phase of the GENOA study (Phase I: 1996-2001), all members of sibships containing ≥ 2 individuals with essential hypertension

clinically diagnosed before age 60 were invited to participate, including both hypertensive and normotensive siblings. Exclusion criteria of the GENOA study were secondary hypertension, alcoholism or drug abuse, pregnancy, insulin-dependent diabetes mellitus, or active malignancy. Eighty percent of African Americans (1,482 subjects) and 75% of non-Hispanic whites (1,213 subjects) from the initial study population returned for the second examination (Phase II: 2001-2005). Study visits were made in the morning after an overnight fast of at least eight hours. Demographic information, medical history, clinical characteristics, lifestyle factors, and blood samples were collected in each phase. Written informed consent was obtained from all subjects and approval was granted by participating institutional review boards.

DNA methylation was measured on the peripheral blood leukocytes of 1,008 African American participants using stored blood samples collected during the Phase II examination. The EZ DNA Methylation Gold Kit (Zymo Research, Irvine CA) was used for bisulfite conversion. The methylation assay was performed at the Mayo Clinic Advanced Genomics Technology Center using Illumina® Infinium HumanMethylation27 BeadChips and the Illumina BeadXpress reader. Seven samples were excluded from analysis due to poor bisulfite conversion efficiency (intensity < 4,000). An additional 28 samples were removed because of poor background signals, leaving a total 973 samples. The *lumi* package[17] in R software was used for background adjustment, color balance adjustment, and quantile normalization. Cell proportions for CD8 T lymphocytes, CD4 T lymphocytes, natural killer cells, B cells, monocytes, granulocytes were estimated using Houseman's method.[2] Detection p-values were calculated for each sample at each CpG site, and values were set as missing when detection P-value was >0.01. A total of 2,302 CpG sites were excluded because >1% of samples had a detection P-value of >0.01. A total of 30 samples with call rate <95% were also removed.

CRP was measured from blood samples obtained during Phase II by a highly sensitive immunoturbidimetric assay.[18] CRP was natural log-transformed. None of the participants had natural log-transformed CRP that was smaller or larger than 4 standard deviations from the mean CRP levels. Two participants with type 1 diabetes were excluded from this analysis, and two participants didn´t have CRP levels available. A total of 939 African American GENOA participants were included in the analyses.

Genetics of Lipid Lowering Drugs and Diet Network (GOLDN)

Genetics of Lipid Lowering Drugs and Diet Network (GOLDN) is a family-based study, described extensively in other publications.[19, 20] We recruited families with at least two siblings where all participants (n=1,327) self-identified as European Americans using the study population of the National Heart, Lung, and Blood Institute Family Heart Study at the Minneapolis and Salt Lake City sites. The study was conducted to identify genetic and epigenetic factors contributing to both lipid-raising (i.e. postprandial lipemia challenge) and lipid-lowering (3 week fenofibrate therapy) interventions. Participants fasted for at least 8 hours, did not drink alcohol for 24 hours, and did not take lipid-lowering medications for 4 weeks before each study visit. We collected DNA samples and measured hsCRP for this epigenome-wide study at the baseline visit, i.e. before the lipid-raising and the lipid-lowering interventions. The study protocol was approved by Institutional Review Boards at the University of Minnesota, University of Utah, Tufts University/New England Medical Center, and University of Alabama at Birmingham; all research procedures heeded the principles of the Declaration of Helsinki and all participants provided written informed consent.

After blood collection, samples were centrifuged at 2000 × *g* for 15 minutes at 4 degrees C within 20 minutes of collection, stored frozen at −70 degrees C, and analyzed at the same time for each participant to eliminate inter-assay variability.[21] HsCRP was measured

on the Hitachi 911 using a latex particle enhanced immunoturbidimetric assay (Kamiya Biomedical Company, Seattle, WA), with a reliability coefficient of 0.99.[21, 22]

The methodology of epigenome-wide studies in GOLDN is described in detail in prior manuscripts from our group.[20, 23] Briefly, we harvested CD4+ T-cells from frozen buffy coat samples from peripheral blood and isolated DNA using antigen-specific magnetic beads (Invitrogen, Carlsbad, CA, USA). We lysed cells captured on the beads, extracted DNA using DNeasy kits (Qiagen, Venlo, Netherlands), and performed bisulfite conversion using the EZ DNA Methylation Kit (Zymo Research, Irvine, CA, USA). We used the Infinium HumanMethylation450 array (Illumina, San Diego, CA) to measure genome-wide DNA methylation. After whole genome amplification, hybridization, and imaging as suggested by the array manufacturer, we used Illumina GenomeStudio software to estimate methylation (β) scores and detection P-values.[20, 23]

During the quality control stage, we excluded: 1) samples that were missing more than 1.5% data points, 2) β scores with an associated detection P-value greater than 0.01, and 3) CpGs where the probe sequence mapped either multiple loci or to a location that did not match the annotation file, or where more than 10% of samples yielded inadequate intensity.[23] After quality control, 991 samples and 461,281 CpGs remained. For normalization, we used the ComBat package (http://www.bu.edu/jlab/wp-assets/ComBat/Download.html) for R software.[23, 24] Adjusting for both plate and position on the plate, we normalized random subsets of 20,000 CpGs per run, with each array of 12 samples used as a “batch.” We also generated principal components (PCs) based on the β scores of all autosomal CpGs that passed quality control to adjust for cell purity.[20, 23] Predicted CD4+ T-cell purity was estimated using a linear model based deconvolution method.[2, 20, 25] We used a reference data set of CD4+ T-cells and granulocytes extracted from fresh blood sample to estimate the percentage of CD4+ T-cells in each sample.[24]

Predicted CD4+ T-cell purity was associated with the first PC ($r2=0.85$) but not other PCs.[20, 26] We modeled associations between methylation scores at each CpG site and age using linear mixed models, adjusted for age, sex, smoking, body mass index, and the first 4 CD4+ T-cell purity PCs as fixed effects, as well as for pedigree as a random effect using the *lmekin* function of the *kinship* package (http://cran.r-project.org/src/contrib/Archive/kinship/) in R.[27] The analysis only included participants with complete covariate information (n=976). The DNA methylation data of the GOLDN study is available on dbGAP (accession number: phs000741.v1.p1).

Grady Trauma Project (GTP)

The Grady Trauma Project (GTP) is a population-based, prospective study of demographic characteristics, trauma exposure, and prevalence of post-traumatic stress disorder and major depressive disorder in an urban, predominantly African-American population.[28] Subjects were recruited prospectively from the waiting rooms of primary care and obstetrics-gynecology clinics of Grady Memorial Hospital in Atlanta, GA. Exclusion criteria included mental retardation, active psychosis, or the inability to give informed consent. Written and verbal informed consent was obtained for all participating subjects. All procedures in this study were approved by the Institutional Review Boards of Emory University School of Medicine and Grady Memorial Hospital. Since its inception in 2005, over 5,000 subjects have been interviewed for the study.

Blood samples were collected and immediately processed and stored at -80C until the time of assay. Serum CRP was measured using an immunoturbidometric assay from Sekisui Diagnostics (www.sekisuidiagnostics.com) on the Beckman AU480 chemistry analyzer, with an inter-assay coefficient of variation (CV) of 5.2% and an intra-assay CV of 3.1%. Serum IL-6 was assayed using a high sensitivity sandwich ELISA by R&D Systems (HS600B;

www.rndsystems.com), with an inter-assay CV of 7.7% and an intra-assay CV of 7.4%. On the same day of sample collection, a trained clinician obtained height and weight measures from individuals. Body mass index was calculated as weight in kilogram divided by height in meters squared. Smoking information was collected from participants using an adapted KMSK questionnaire tool. This tool (originally described in Kellogg et al. 2003[29]) records, using a numerical scale, the current frequency of smoking, the duration of time that this frequency has been maintained and the amount of cigarettes smoked during this period. The adapted tool used in GTP recorded this information for both the 30 days prior and the time period where participant smoking was greatest for 425 individuals. Frequency (coded on a 0-5 point scale, where 5 = smoking at regular intervals most/all days; 4 = smoking at specific times of day most/all days; 3 = once a day most/all days; 2 = 20-100 times in lifetime; 1 = less than 20 times in lifetime; 0 = never smoked) for both time periods (hereafter referred to as '30-day' and 'maximum') was used to create a variable describing whether the individual is a current, former or never smoker (CFN). The CFN scale was determined as follows: 1) An individual was classified as a current smoker (N = 101), if their 30-day frequency was coded as a 3, 4 or 5 and their maximum frequency was coded as a 3, 4, 5 or missing. 2) An individual was classified as a former smoker (N = 71), if 30-day frequency was coded as a 0, 1, or 2, and maximum frequency was coded as a 3, 4 or 5. 3) An individual was classified as a never smoker (N = 136), if their maximum frequency was coded as a 0,1, or 2 and their 30-day frequency was coded as a 0, 1, 2 or missing. If an individual did not meet the above criteria (N = 52) or did not supply any smoking information (N = 65), their score on the CFN scale was recorded as missing.

For the methylation analysis, we extracted DNA from whole blood at the Max Planck Institute in Munich using the Gentra Puregene Kit (Qiagen). Genomic DNA was then bisulfite converted using the Zymo EZ-96 DNA Methylation Kit (Zymo Research). We assessed DNA

methylation at >480,000 CpG sites using Illumina HumanMethylation450 BeadChip arrays, with hybridization and processing performed according to the instructions of the manufacturer. For each CpG site and individual, we collected two data points: M (the total methylated signal), and U (the total unmethylated signal). We set to missing data points with 1) a detection p-value greater than 0.001 or 2) a combined signal less than 25% of the total median signal and less than both the median unmethylated and median methylated signal. We removed individual samples from analysis if they were outliers in a hierarchical clustering analysis or had 1) a mean total signal less than half of the median of the overall mean signal or 2000 arbitrary units, or 2) a missingness rate above 5%. Similarly, we removed from analysis CpG sites with a missingness rate above 10%. We then computed β-values for each individual at each CpG site as the total methylated signal divided by the total signal:

$$\beta = \frac{M}{U + M}$$

The DNA methylation data from the GTP study is available on GEO (accession number: GSE72680).

The Invecchiare in Chianti Study (InCHIANTI)

The InCHIANTI study is a population-based epidemiological study aimed at evaluating the factors that influence mobility in the older population living in the Chianti region in Tuscany, Italy. The details of the study have been previously reported.[30] Briefly, 1,616 residents were selected from the population registry of Greve in Chianti (a rural area: 11,709 residents with 19.3% of the population greater than 65 years of age), and Bagno a Ripoli (Antella village near Florence; 4,704 inhabitants, with 20.3% greater than 65 years of age). The participation rate was 90% (n=1,453), and the subjects ranged between 21-102 years of age. The study protocol was approved by the Italian National Institute of Research and Care of Aging Institutional Review and Medstar Research Institute (Baltimore, MD). This analysis was

restricted to 498 subjects with data on DNA methylation and C-reactive protein (CRP) measures. Serum high sensitivity CRP was measured using ELISA and colorimetric competitive immunoassay (Roche Diagnostics, GmbH, Mannheim, Germany). Assessment of DNA methylation has been previously described.[31] Briefly, DNA was extracted from buffy coat samples and bisulfite converted using Zymo EZ-96 DNA Methylation kit (Zymo Research Corp., Irvine, CA) and methylation status of 485,577 CpG sites was assessed with Illumina Infinium HumanMethylation450 BeadChip (Illumina Inc, San Diego, CA). Quality control filtering and normalization accomplished using the wateRmelon package.[15] Markers were excluded if the bead count was less than 3 in ≥ 5% of samples. Samples and markers where also excluded if ≥ 5% of detection p-values were greater than 0.01. A background adjustment and quantile normalization were applied to the filtered data set; the selected method normalizes both methylated and unmethylated probes as well as type I and II assays separately. Locations were annotated using the FDb.InfiniumMethylation.hg19 database. Methylation markers on the X and Y chromosome, as well as markers with potentially cross-reactive probes and probes that may be polymorphic in European populations (AF ≥ .01) were excluded from analyses.

Kooperative Gesundheitsforschung in der Region Augsburg (KORA)

The Cooperative Health Research in the Region of Augsburg (KORA) study is a series of independent surveys and follow-up studies from the general population living in the region of Augsburg, Southern Germany. All participants are residents of German nationality identified through the local registration office. The study was approved by the ethics committee of the Bavarian Medical Association, and informed written consent was obtained from all participants.[32] For the present study, blood was collected in fasting subjects of the KORA F4 subjects without stasis and kept at 4°C until centrifugation. Plasma concentrations of high-

sensitivity CRP were assessed by an immunonephelometric assay on a BNII analyzer (Dade Behring, Marburg, Germany).

DNA methylation measurements were performed on whole blood samples of the KORA F4 study using Illumina HM450k Bead Chip arrays and data were preprocessed as described previously.[33, 34] Briefly, genomic DNA was bisulfite converted, amplified and enzymatically fragmented. After application of samples the arrays were fluorescently stained and scanned using Illunina HiScan SQ scanner. Raw methylation values were pre-processed using R, version 3.0.1, partly following a pipeline developed by Touleimat and Tost.[35] Samples with more than 20% low-confidence probes (defined as probes with signals summarized from less than three functional beads, and probes with a detection p-values larger than 0.01) and CpG sites representing or being located in 50 bp proximity to single nucleotide polymorphisms (SNPs) with a minor allele frequency of at least 5% were excluded from the data set. Color bias adjustment and background correction based on negative control probes were conducted (R package *lumi*, version 2.12.0).[17] Beta-values from low-confidence probes were set to missing and CpG sites with more than 5% low-confidence probes were removed from the data set.[35] Finally, beta-mixture quantile normalization (BMIQ)[1] was applied to the DNA methylation data (R package 'wateRmelon', version 1.0.3). DNA methylation data was available in 1,799 subjects. For the current analysis, 88 individuals were excluded since they used anti-inflammatory medication, 3 individuals had missing CRP values, and 8 had one or more missing covariate. Finally, a total of 1,700 individuals with CRP, DNA methylation data and covariates available were included in the present analyses.

Lothian Birth Cohorts (LBC1921/1936)

The Lothian Birth Cohorts of 1921 and 1936 are two longitudinal studies of ageing.[36-38] They derive from the Scottish Mental Surveys of 1932 and 1947, respectively, when nearly all

11 year old children in Scotland completed a test of general cognitive ability.[38] Survivors living in the Lothian area of Scotland were recruited in late-life at mean age 79 for LBC1921 (n=550) and mean age 70 for LBC1936 (n=1,091). Follow-up has taken place at ages 70, 73, and 76 in LBC1936 and ages 79, 83, 87, and 90 in LBC1921. Collected data include genetic information, longitudinal epigenetic information, longitudinal brain imaging (LBC1936), and numerous blood biomarkers, anthropomorphic and lifestyle measures. Post QC, DNA methylation data were available on 299 LBC1936 participants at age 73, and for 175 LBC1921 participants at age 87. Finally, 169 LBC1921 and 296 LBC1936 participants were included with DNA methylation data, CRP levels, and covariate information available.

Detailed information about the collection and QC steps undertaken on the LBC methylation data have been reported previously.[39] Briefly, the Infinium HumanMethylation450 BeadChip (Illumina Inc, San Diego, CA) was used to measure DNA methylation in whole blood of consenting participants. Background correction was performed and QC was used to remove probes with a low detection rate, low quality (manual inspection), low call rate, and samples with a poor match between genotypes and SNP control probes, and incorrect predicted sex. A total number of 450,712 probes passed QC.

At the first and second LBC1936 visits, and the third LBC1921 visit, non-fasting blood samples were collected. At LBC1936 wave 1, a CRP assay was performed using a dry slide immuno-rate method on the OrthoFusion 5.1 F.S analyzers. At wave 3 of LBC1921, CRP was measured using using high-sensitivity ELISA kits (R&D Systems, Oxon, UK). At each wave of the respective studies, basic anthropometric measures were taken, including height and weight. Body mass index was calculated as weight in kilogram divided by height in metres squared. A self-reported measure of smoking behaviour(current, former and never) was obtained at each of the study waves. White blood cell counts (eosinophils, basophils, neutrophils, lymphocytes, and monocytes) were also measured at each wave.[40] The DNA

methylation data from the LBC cohorts is available in European Genome-phenome Archive (accession number: EGAS00001000910).

Normative Aging Study (NAS)

The US Department of Veterans Affairs (VA) Normative Aging Study (NAS) is an ongoing longitudinal cohort of aging men established in 1963. Participants were 21-80 years of age and free of known chronic medical conditions at enrollment.[41] Clinical health data and demographic factors are collected at 3-5 year intervals and supplemented with periodic medical examinations. DNA samples were collected from the 675 active participants in the period 1999-2007 and used in DNA methylation analysis. From the available methylation samples, we excluded participants who were non-whites or had missing information on covariates, leaving a total of 651 participants, out of those 648 had C-reactive protein (CRP) values.

The NAS study was approved by the Institutional Review Boards (IRBs) of the participating institutions. Participants have provided written informed consent at each visit. DNA was extracted from buffy coat using the QIAamp DNA Blood Kit (QIAGEN, Valencia, CA). 500 ng of DNA was used to perform bisulfite conversion using the EZ-96 DNA Methylation Kit (Zymo Research, Orange, CA). To reduce the chip and plate effects, we used a two-stage age-stratified algorithm to randomize samples and ensure similar age distributions across chips and plates; 12 samples – which were sampled across all the age quartiles – were randomized to each chip, then chips were randomized to plates (each housing eight chips). Quality control analysis was performed to remove samples and probes where >1% of probes and samples, respectively, had a detection p-value > 0.05. The remaining samples were preprocessed using the Illumina-type background correction[42] and normalized with the dye-

bias and BMIQ[1] adjustments, which were used to generate beta methylation values. 477,928 CpG probes were in the working set.

At each study visit, fasting blood samples were collected form each participant. C-reactive protein (CRP) for all time points was analyzed on archived samples stored at -80° Celsius. Samples from each participant were analyzed in duplicate and in a single batch to avoid between-batch analytical variation. The performance of the assays was monitored with standard quality control procedures including the analysis of quality control samples in each batch.

CRP was measured in serum using immunoturbidimetric assays on a Hitachi 917 analyzer (Roche Diagnostics, Indianapolis, IN, USA) with reagents and calibrators from Denka Seiken (Niigata, Japan).[43]

At each in-person examination visit, participants provided demographic, lifestyle and anthropometric information, e.g. age (continuous), smoking status (never/former/current), pack-years (continuous), and Body Mass Index (BMI) (continuous – Kg/m^2). At each examination visit from fasting blood drawn samples we measured white blood cells counts (neutrophils, lymphocytes, monocytes, eosinophils, basophils). DNA methylation data from the NAS study is available on dbGAP (accession number: phs000853.v1.p1).

Rotterdam Study (RS)

The Rotterdam Study is a prospective population based cohort study in a well-defined area of Rotterdam, the Netherlands. The design of the Rotterdam Study has been detailed elsewhere.[44] For the current analysis we used data from individuals aged 45 years and older that participated in the third cohort of the Rotterdam Study. In the first visit of the third cohort, 3,934 participants were examined between February 2006 and December 2008. Whole

blood DNA methylation was quantified in a random subset of 750 individuals with genotyping and RNA expression data available.

DNA was extracted from whole peripheral blood (stored in EDTA tubes) by standardized salting out methods. Genome-wide DNA-methylation levels in 750 subjects from the Rotterdam Study-III were determined using the Illumina HumanMethylation 450K beadarray (Illumina, Inc., San Diego, CA, USA). In short, samples (500ng of DNA per sample) were first bisulfite treated using the Zymo EZ-96 DNA-methylation kit (Zymo Research, Irvine, CA, USA). Next, they were hybridized to the arrays according to the manufacturers protocol. During quality control samples showing incomplete bisulfite treatment were excluded (n=5) as were samples with a low detection rate (<99%) (n=7), or gender swaps (n=4), leaving 734 individuals. Probes with a detection p-value>0.01 in >1% samples, were filtered out. A total number of 474,528 probes passed the quality control and the filtered β values were normalized with DASEN implemented in the *wateRmelon* package in R statistical software.[15]

At the first center visit, fasting blood samples were collected. The samples were immediately put on ice and were processed within 30 minutes after which the samples were kept frozen at -80 °C until the measurement of high-sensitivity CRP (hs-CRP) in January 2012. Serum CRP was measured by a particle enhanced immunoturbidimetric assay (Roche Diagnostics GmnH, Mannheim, Germany). This assay measures CRP values ranging from 0.3-350 mg/L. From the 734 available methylation samples, after excluding individuals with auto-immune diseases and individuals using immune-modulating agents, the total number of participants with serum CRP levels and DNA methylation measurement was 702.

During the research center visit, anthropometric measures including height and weight were obtained. Body mass index was calculated as weight in kilogram divided by height in meters squared. Smoking behavior (current, former and never) was assessed during home

interview by trained research assistants. White blood cells counts (monocytes, granulocytes and lymphocytes) were measured immediately at the research center using a standard hematology analyzer (Beckman Coulter, Pasadena, CA, USA).

Women's Health Initiative (WHI)

Women were selected from one of two WHI large sub cohorts that had previously undergone genome wide genotyping as well as profiling for 7 cardiovascular disease related biomarkers including total cholesterol, HDL, LDL, triglycerides, CRP, creatinine, insulin, and glucose through 2 core WHI ancillary studies. The first cohort is the WHI SNP Health Association Resource (SHARe) cohort of minorities that includes >8000 African American (AA) women and >3500 Hispanic women. These women were genotyped through WHI core study M5-SHARe (www.whi.org/researchers/data/WHIStudies/StudySites/M5) and underwent biomarker profile through WHI Core study W54-SHARe (...data/WHIStudies/StudySites/W54). The second cohort consists of a combination of European Americans (EA) from the two Hormonal Therapy (HT) trials selected for GWAS and biomarkers in core studies W58 (.../data /WHIStudies/StudySites/W58) and W63 (.../data/WHIStudies/StudySites/W63). From these two cohorts, two sample sets were formed. The first (sample set 1) is a sample set of 637 CHD cases and 631 non-CHD cases as of Sept 30, 2010. The second sample set (sample set 2) is a non-overlapping sample of 432 cases of CHD and 472 non-cases as of September 17, 2012. All women with measures of inflammation that passed QC were included in this analysis.

DNA methylation data from whole blood. Methylation analysis was performed at HudsonAlpha Institute of Biotechnology using the Illumina Infinium HumanMethylation450 BeadChip. The Illumina BeadChips measures bisulfite-conversion-based, single-CpG resolution DNA methylation levels at 485,577 different CpG sites in the

human genome. These data were generated by following the standard protocol of Illumina methylation assays, which quantifies methylation levels by the β value using the ratio of intensities between methylated and un-methylated alleles. Beta scores were generated using Illumina's GenomeStudio software without any normalization or background subtraction options. Any value with a detection p-value above 0.01 was set to missing, and samples with more than 1.5% missing data were removed. Additionally, CpGs with greater than 10% missing data were removed. Batch normalization was carried out using non-parametric empirical Bayes normalization using the Combat function in R,[24] using a set of 12 samples on a single array as a batch. Normalization was performed using parallel operations on random subsets of 20,000 CpGs to improve computational efficiency. Probes using Infinium I chemistry were normalized separately from those using Infinium II chemistry, and a chemistry correction was applied after normalization. The chemistry correction was based on applying a second-order polynomial fit using CpGs of differing chemistries within 50 bp of each other (correlations > 0.99 for CpG pairs within this distance).[23] The DNA methylation data from the WHI study will become available on dbGAP (accession number: phs000200.v10.p3). In total, after excluding individuals using anti-inflammatory medication and auto-immune diseases, 471 European CHD controls, 478 European CHD cases, 309 African CHD controls, and 294 African CHD cases were included in the analyses with DNA methylation, CRP levels, and covariate information available.

High-sensitive CRP (hsCRP) was measured in serum or plasma using a latex-particle enhanced immunoturbidimetric assay kit (Roche Diagnostics, Indianapolis, IN 46250) and read on the Roche Modular P Chemistry analyzer (Roche Diagnostics). The reference range is 0 – 5 mg/L. The inter-assay CV in our laboratory is 4.5%.

b. Genotyping information

Framingham Heart Study

Genotype data were obtained from buffy coat samples, assayed using the MIPS 50K and Affymetrix 500K platforms for a total of 549,781 SNPs. Of these genotyped SNPs, 137,728 genotyped SNPs were removed based on the following filtering criteria: 22,018 SNPs for HWE p-value<1e-6, 48,285 SNPs for a call rate<96.9%, 66,063 SNPs for a MAF < 0.01, 82 SNPs due to not mapping correctly from Build 36 to Build 37 locations, 428 SNPs missing a physical location, 25 SNPs for excessive Mendelian errors, 786 SNPs due to not being on chromosomes 1-22 or X, and 41 duplicate SNPs. A total of 412,053 genotyped SNPs after QC were used as input to the genotype imputation package Minimac4. Minimac's GIANT 1000G Imputation protocol using "Cosmopolitan SNP set" was used, with the SNP phasing options of: -rounds 20 -states 200 -phase -sample 5, yielding a total of 39,315,185 SNPs.

Rotterdam Study

The Illumina 610 quad was used for genotyping in 3,540 individuals from the third cohort of the Rotterdam Study. Genotyping was successful in 3,361 individuals with a sample call rate >97.5%. SNPs with a call rate <95% and HWE p<10-6 were excluded. The final dataset comprised data on 543,360 SNPs in 3,054 participants. Imputation was conducted using the algorithm implemented in MACH. To obtain imputed data, more restrictive SNP filters including a minor allele frequency >0.01, SNP call rate >0.98, and HWE p-value $>1\times10^{-6}$ were applied and 514,073 passed the filters. In total 2,543,887 SNPs were imputed using phased haplotypes of HapMap CEU trios.

c. Gene expression quantification

GTP

Whole blood was collected between 8 - 9 a.m. in Tempus RNA tubes for 398 subjects. All subjects were instructed to fast before blood collection. Whole genome expression profiles were generated for 398 subjects at the Max-Planck Institute as follows: RNA was isolated using the Versagene kit (Gentra Systems, Minneapolis, U.S.A.), quantified using the Nanophotometer and quality checks were performed on the Agilent Bioanalyzer. 250 nanograms of total RNA were reverse transcribed to cDNA, converted to cRNA and biotin-labeled using the Ambion kit (AMIL1791, Applied Biosystems), 750 nanograms of cRNA were hybridized to Illumina HT-12 v3.0 or v4.0 arrays (Illumina, San Diego, California, U.S.A) and incubated overnight for 16 hours at 55ºC. Arrays were washed, stained with Cy3 labeled streptavidin, dried and scanned on the Illumina BeadScan confocal laser scanner. 21,394 transcripts were on both the v3.0 and v4.0 arrays and were significantly expressed above background levels (detection $P<.05$) in at least 10% of subjects, and were thus eligible for further analysis. Of the initial 398 subjects, 359 subjects with self-reported African-American ancestry were selected. Age of subjects at blood draw ranged from 16-78 years.

FHS

In the FHS, the methylation-expression analysis was performed on 2,262 individuals having both methylation and gene expression data. The methylation data were as previously described. The expression data were obtained from whole blood samples processed using the PaxGene tubes and assayed using the Affymetrix Human Exon Array ST-1.0. The details of sample preprocessing was previously detailed.[45] The FHS gene expression data is avalaible on dbGAP (accession number: phs000363.v15.p10).

InCHIANTI

The InCHIANTI microarray dataset comprises expression data from peripheral blood drawn from 695 subjects aged 30 to 104 years between 2007/2008 (the same blood draw as the DNA methylation data), described previously.[46] Briefly, blood was collected into PAXgene tubes (BD Biosciences), and extracted using the PAXgene blood RNA kit (Qiagen, Paisley, UK). Microarray analysis was carried out on the Illumina HT-12 v3 array (Illumina, San Diego, USA). The expression data were quantile normalized, log2 transformed and the probe and sample means were centered to zero. The data has been deposited in the GEO public database (accession number: GSE48152).

KORA

Gene expression profiling was performed in a random subsample of the KORA S4 study comprising of 1,002 elderly individuals aged 62 to 81 years[47] using the Illumina HumanHT-12 v3 BeadChip as described elsewhere.[48] Briefly, after isolating RNA from whole blood (PAXgene Blood miRNA Kit, Qiagen, Hilden, Germany) and assessing its purity and integrity (Agilent Bioanalyzer with the 6000 Nano LabChip reagent set, Agilent Technologies, Germany) the RNA was reversely transcribed (TotalPrep-96 RNA Amp Kit, Ambion, Germany) and hybridized to the Illumina HumanHT-12 v3 Expression BeadChip. Missing values were imputed (Illumina Software GenomeStudio). The raw expression data were quantile normalized, log2 transformed and the probe and sample means were centered to zero. Gene expression data are available on ArrayExpress (E-MTAB-1708). Expression analyses in the KORA study was carried out in 707 individuals with both, quality controlled gene expression and methylation data.

Rotterdam Study

Whole-blood was collected (PAXGene Tubes – Becton Dickinson) and total RNA was isolated (PAXGene Blood RNA kits - Qiagen). To ensure a constant high quality of the RNA preparations, all RNA samples were analysed using the Labchip GX (Calliper) according to the manufacturer's instructions. Samples with an RNA Quality Score more than 7 were amplified and labelled (Ambion TotalPrep RNA), and hybridized to the Illumina HumanHT12v4 Expression Beadchips as described by the manufacturer's protocol. Processing of the Rotterdam Study RNA samples was performed at the Genetic Laboratory of Internal Medicine, Erasmus University Medical Centre Rotterdam. The RS-III expression dataset is available at GEO (Gene Expression Omnibus) public repository under the accession number GSE33828. Illumina gene expression data was quantile-normalized to the median distribution and subsequently log2-transformed. The probe and sample means were centered to zero. Genes were declared significantly expressed when the detection p-values calculated by GenomeStudio were less than 0.05 in more than 10% of all discovery samples, which added to a total number of 21,238 probes.[49] Quality control was done using the eQTL-mapping pipeline. We only analyzed probes that uniquely mapped to the human genome build 37.[48]

References

1. Teschendorff AE, Jones A, Fiegl H, Sargent A, Zhuang JJ, Kitchener HC, Widschwendter M: **Epigenetic variability in cells of normal cytology is associated with the risk of future morphological transformation.** *Genome Med* 2012, **4:**24.
2. Houseman EA, Accomando WP, Koestler DC, Christensen BC, Marsit CJ, Nelson HH, Wiencke JK, Kelsey KT: **DNA methylation arrays as surrogate measures of cell mixture distribution.** *BMC bioinformatics* 2012, **13:**86.
3. Fried LP, Borhani NO, Enright P, Furberg CD, Gardin JM, Kronmal RA, Kuller LH, Manolio TA, Mittelmark MB, Newman A: **The cardiovascular health study: design and rationale.** *Annals of epidemiology* 1991, **1:**263-276.
4. Aryee MJ, Jaffe AE, Corrada-Bravo H, Ladd-Acosta C, Feinberg AP, Hansen KD, Irizarry RA: **Minfi: a flexible and comprehensive Bioconductor package for the analysis of Infinium DNA methylation microarrays.** *Bioinformatics* 2014, **30:**1363-1369.
5. Maksimovic J, Gordon L, Oshlack A: **SWAN: Subset-quantile within array normalization for illumina infinium HumanMethylation450 BeadChips.** *Genome Biol* 2012, **13:**R44.
6. Fortin J-P, Labbe A, Lemire M, Zanke BW, Hudson TJ, Fertig EJ, Greenwood CMT, Hansen KD: **Functional normalization of 450k methylation array data improves replication in large cancer studies.** *Genome biology* 2014, **15:**503.
7. Macy EM, Hayes TE, Tracy RP: **Variability in the measurement of C-reactive protein in healthy subjects: implications for reference intervals and epidemiological applications.** *Clinical chemistry* 1997, **43:**52-58.
8. Day N, Oakes S, Luben R, Khaw KT, Bingham S, Welch A, Wareham N: **EPIC-Norfolk: study design and characteristics of the cohort. European Prospective Investigation of Cancer.** *British journal of cancer* 1999, **80:**95-103.
9. Bendinelli B, Masala G, Saieva C, Salvini S, Calonico C, Sacerdote C, Agnoli C, Grioni S, Frasca G, Mattiello A: **Fruit, vegetables, and olive oil and risk of coronary heart disease in Italian women: the EPICOR Study.** *The American journal of clinical nutrition* 2010**:**ajcn. 000521.
10. Fiorito G, Guarrera S, Valle C, Ricceri F, Russo A, Grioni S, Mattiello A, Di Gaetano C, Rosa F, Modica F: **B-vitamins intake, DNA-methylation of One Carbon Metabolism and homocysteine pathway genes and myocardial infarction risk: the EPICOR study.** *Nutrition, metabolism, and cardiovascular diseases: NMCD* 2014, **24:**483-488.
11. Marabita F, Almgren M, Lindholm ME, Ruhrmann S, Fagerström-Billai F, Jagodic M, Sundberg CJ, Ekström TJ, Teschendorff AE, Tegnér J: **An evaluation of analysis pipelines for DNA methylation profiling using the Illumina HumanMethylation450 BeadChip platform.** *Epigenetics* 2013, **8:**333-346.
12. Feinleib M, Kannel WB, Garrison RJ, McNamara PM, Castelli WP: **The Framingham offspring study. Design and preliminary data.** *Preventive medicine* 1975, **4:**518-525.
13. Kannel WB, Feinleib M, McNamara PM, Garrison RJ, Castelli WP: **An investigation of coronary heart disease in families The Framingham offspring study.** *American journal of epidemiology* 1979, **110:**281-290.
14. Kathiresan S, Larson MG, Vasan RS, Guo C-Y, Gona P, Keaney JF, Wilson PWF, Newton-Cheh C, Musone SL, Camargo AL: **Contribution of clinical correlates and 13 C-reactive protein gene polymorphisms to interindividual variability in serum C-reactive protein level.** *Circulation* 2006, **113:**1415-1423.
15. Pidsley R, Wong CCY, Volta M, Lunnon K, Mill J, Schalkwyk LC: **A data-driven approach to preprocessing Illumina 450K methylation array data.** *BMC genomics* 2013, **14:**293.

16. Daniels PR, Kardia SLR, Hanis CL, Brown CA, Hutchinson R, Boerwinkle E, Turner ST: **Familial aggregation of hypertension treatment and control in the Genetic Epidemiology Network of Arteriopathy (GENOA) study.** *The American journal of medicine* 2004, **116:**676-681.
17. Du P, Kibbe WA, Lin SM: **lumi: a pipeline for processing Illumina microarray.** *Bioinformatics* 2008, **24:**1547-1548.
18. Kullo IJ, Seward JB, Bailey KR, Bielak LF, Grossardt BR, Sheedy PF, Peyser PA, Turner ST: **C-reactive protein is related to arterial wave reflection and stiffness in asymptomatic subjects from the community.** *American journal of hypertension* 2005, **18:**1123-1129.
19. Corella D, Arnett DK, Tsai MY, Kabagambe EK, Peacock JM, Hixson JE, Straka RJ, Province M, Lai C-Q, Parnell LD: **The− 256T> C polymorphism in the apolipoprotein A-II gene promoter is associated with body mass index and food intake in the genetics of lipid lowering drugs and diet network study.** *Clinical chemistry* 2007, **53:**1144-1152.
20. Aslibekyan S, Kabagambe EK, Irvin MR, Straka RJ, Borecki IB, Tiwari HK, Tsai MY, Hopkins PN, Shen J, Lai C-Q: **A genome-wide association study of inflammatory biomarker changes in response to fenofibrate treatment in the genetics of lipid lowering drug and diet network (GOLDN).** *Pharmacogenetics and genomics* 2012, **22:**191.
21. Kabagambe EK, Ordovas JM, Tsai MY, Borecki IB, Hopkins PN, Glasser SP, Arnett DK: **Smoking, inflammatory patterns and postprandial hypertriglyceridemia.** *Atherosclerosis* 2009, **203:**633-639.
22. Aslibekyan S DE, Mendelson M, Zhi D, Guan W, Liang L, et al.: **Epigenome-wide study identified novel methylation loci associated with body mass index and waist circumference.** *Obesity* 2015, **in press**.
23. Absher DM, Li X, Waite LL, Gibson A, Roberts K, Edberg J, Chatham WW, Kimberly RP: **Genome-wide DNA methylation analysis of systemic lupus erythematosus reveals persistent hypomethylation of interferon genes and compositional changes to CD4+ T-cell populations.** *PLoS genetics* 2013, **9:**e1003678.
24. Johnson WE, Li C, Rabinovic A: **Adjusting batch effects in microarray expression data using empirical Bayes methods.** *Biostatistics* 2007, **8:**118-127.
25. Abbas AR, Wolslegel K, Seshasayee D, Modrusan Z, Clark HF: **Deconvolution of blood microarray data identifies cellular activation patterns in systemic lupus erythematosus.** *PloS one* 2009, **4:**e6098.
26. Irvin MR, Zhi D, Joehanes R, Mendelson M, Aslibekyan S, Claas SA, Thibeault KS, Patel N, Day K, Jones LW: **Epigenome-wide association study of fasting blood lipids in the genetics of lipid-lowering drugs and diet network study.** *Circulation* 2014, **130:**565-572.
27. Therneau T: **On mixed-effect Cox models, sparse matrices, and modeling data from large pedigrees.** *URL http://www mayo edu/biostatistics* 2003.
28. Gillespie CF, Bradley B, Mercer K, Smith AK, Conneely K, Gapen M, Weiss T, Schwartz AC, Cubells JF, Ressler KJ: **Trauma exposure and stress-related disorders in inner city primary care patients.** *General hospital psychiatry* 2009, **31:**505-514.
29. Kellogg SH, McHugh PF, Bell K, Schluger JH, Schluger RP, LaForge KS, Ho A, Kreek MJ: **The Kreek–McHugh–Schluger–Kellogg scale: a new, rapid method for quantifying substance abuse and its possible applications.** *Drug and alcohol dependence* 2003, **69:**137-150.
30. Ferrucci L, Bandinelli S, Benvenuti E, Iorio A, Macchi C, Harris TB, Guralnik JM: **Subsystems contributing to the decline in ability to walk: bridging the gap between epidemiology and geriatric practice in the InCHIANTI study.** *Journal of the American Geriatrics Society* 2000, **48:**1618-1625.
31. Moore AZ, Hernandez DG, Tanaka T, Pilling LC, Nalls MA, Bandinelli S, Singleton AB, Ferrucci L: **Change in Epigenome-Wide DNA Methylation Over 9 Years and Subsequent Mortality: Results**

From the InCHIANTI Study. *The Journals of Gerontology Series A: Biological Sciences and Medical Sciences* 2015**:**glv118.
32. Wichmann H, Gieger C, Illig T: **KORA-gen-resource for population genetics, controls and a broad spectrum of disease phenotypes.** *Gesundheitswesen* 2005, **67:**S26.
33. Wahl S, Fenske N, Zeilinger S, Suhre K, Gieger C, Waldenberger M, Grallert H, Schmid M: **On the potential of models for location and scale for genome-wide DNA methylation data.** *BMC bioinformatics* 2014, **15:**232.
34. Pfeifferm L, Wahl S, Pilling LC, Reischl E, Sandling JK, Kunze S, Holdt LM, Kretschmer A, Schramm K, Adamski J: **DNA Methylation of Lipid-Related Genes Affects Blood Lipid Levels.** *Circulation: Cardiovascular Genetics* 2015**:**CIRCGENETICS. 114.000804.
35. Touleimat N, Tost J: **Complete pipeline for Infinium® Human Methylation 450K BeadChip data processing using subset quantile normalization for accurate DNA methylation estimation.** *Epigenomics* 2012, **4:**325-341.
36. Deary IJ, Gow AJ, Pattie A, Starr JM: **Cohort profile: the Lothian Birth Cohorts of 1921 and 1936.** *International journal of epidemiology* 2011**:**dyr197.
37. Deary IJ, Gow AJ, Taylor MD, Corley J, Brett C, Wilson V, Campbell H, Whalley LJ, Visscher PM, Porteous DJ: **The Lothian Birth Cohort 1936: a study to examine influences on cognitive ageing from age 11 to age 70 and beyond.** *BMC geriatrics* 2007, **7:**28.
38. Deary IJ, Whiteman MC, Starr JM, Whalley LJ, Fox HC: **The impact of childhood intelligence on later life: following up the Scottish mental surveys of 1932 and 1947.** *Journal of personality and social psychology* 2004, **86:**130.
39. Shah S, McRae AF, Marioni RE, Harris SE, Gibson J, Henders AK, Redmond P, Cox SR, Pattie A, Corley J: **Genetic and environmental exposures constrain epigenetic drift over the human life course.** *Genome research* 2014, **24:**1725-1733.
40. McIlhagger R, Gow AJ, Brett CE, Corley J, Taylor M, Deary IJ, Starr JM: **Differences in the haematological profile of healthy 70 year old men and women: normal ranges with confirmatory factor analysis.** *BMC Hematology* 2010, **10:**4.
41. Bell B, Rose CL, Damon A: **The Veterans Administration longitudinal study of healthy aging.** *The Gerontologist* 1966, **6:**179-184.
42. Triche TJ, Weisenberger DJ, Van Den Berg D, Laird PW, Siegmund KD: **Low-level processing of Illumina Infinium DNA methylation beadarrays.** *Nucleic acids research* 2013, **41:**e90-e90.
43. McCracken J, Baccarelli A, Hoxha M, Dioni L, Melly S, Coull B, Suh H, Vokonas P, Schwartz J: **Annual ambient black carbon associated with shorter telomeres in elderly men: Veterans Affairs Normative Aging Study.** 2010.
44. Hofman A, Murad SD, van Duijn CM, Franco OH, Goedegebure A, Ikram MA, Klaver CCW, Nijsten TEC, Peeters RP, Stricker BHC: **The Rotterdam Study: 2014 objectives and design update.** *European journal of epidemiology* 2013, **28:**889-926.
45. Joehanes R, Ying S, Huan T, Johnson AD, Raghavachari N, Wang R, Liu P, Woodhouse KA, Sen SK, Tanriverdi K: **Gene expression signatures of coronary heart disease.** *Arteriosclerosis, thrombosis, and vascular biology* 2013, **33:**1418-1426.
46. Harries LW, Hernandez D, Henley W, Wood AR, Holly AC, Bradley-Smith RM, Yaghootkar H, Dutta A, Murray A, Frayling TM: **Human aging is characterized by focused changes in gene expression and deregulation of alternative splicing.** *Aging cell* 2011, **10:**868-878.
47. Rathmann W, Strassburger K, Heier M, Holle R, Thorand B, Giani G, Meisinger C: **Incidence of Type 2 diabetes in the elderly German population and the effect of clinical and lifestyle risk factors: KORA S4/F4 cohort study.** *Diabetic Medicine* 2009, **26:**1212-1219.
48. Schurmann C, Heim K, Schillert A, Blankenberg S, Carstensen M, Dörr M, Endlich K, Felix SB, Gieger C, Grallert H: **Analyzing illumina gene expression microarray data from different tissues:**

methodological aspects of data analysis in the metaxpress consortium. *PloS one* 2012, **7:**e50938.
49. Westra H-J, Peters MJ, Esko T, Yaghootkar H, Schurmann C, Kettunen J, Christiansen MW, Fairfax BP, Schramm K, Powell JE: **Systematic identification of trans eQTLs as putative drivers of known disease associations.** *Nature genetics* 2013, **45:**1238-1243.
